# Intelligent Wearable Rehabilitation System Based on Edge Computing

**DOI:** 10.3390/s26185755

**Published:** 2026-09-10

**Authors:** Chiung-Hsing Chen, Yi-Chen Wu, Jwu-Jenq Chen, Yu-Chen Lin

**Affiliations:** 1 Department of Telecommunication Engineering, National Kaohsiung University of Science and Technology, Kaohsiung 811213, Taiwan; chiung@nkust.edu.tw (C.-H.C.); c108185117@nkust.edu.tw (Y.-C.L.); 2 Department of Computer Science and Information Engineering, Chang Jung Christian University, Tainan 711301, Taiwan

**Keywords:** self-rehabilitation, edge computing, STM32, NanoEdge AI, database

## Abstract

Colles fracture is a common type of wrist fracture, typically resulting from falling onto an outstretched hand. Postoperative patients often require long-term self-rehabilitation to restore wrist function. However, traditional rehabilitation relies on medical personnel and lacks real-time feedback and progress tracking, which may lead to poor rehabilitation or even deterioration of the condition. This article proposes an intelligent wearable system based on edge computing to enhance the efficiency of self-rehabilitation, improve system portability, and ensure comprehensive recording of rehabilitation data. The system performs real-time data processing and feedback to assist patients and healthcare providers in monitoring rehabilitation progress and optimizing recovery outcomes. The proposed system integrates an STM32 microcontroller, NanoEdge AI, and a 9-axis inertial sensor to detect and evaluate the accuracy of hand rehabilitation movements, ensuring the precision of self-rehabilitation. All rehabilitation movements are schemed under the guidance of professional physicians to ensure correctness and minimize the risk of secondary injuries caused by improper rehabilitation. Data such as motion records, training duration, and count are transmitted via Bluetooth to the Local database for further analysis. A custom web interface allows healthcare providers to monitor and analyze collected data. This approach improves diagnostic accuracy, supports personalized rehabilitation recommendations, and improves treatment outcomes and patient recovery success rates.

## 1. Introduction

In recent years, with the aging of the population, the incidence of fractures caused by falls has continued to increase. Among these, the Colles fracture, which typically results from falling onto an outstretched hand, has become one of the most common types [1]. This fracture is closely associated with osteoporosis [2], and the majority of patients are women over the age of 60 [3]. After undergoing surgical or immobilization treatment, patients often experience complications such as decreased wrist mobility and muscle weakness, which require long-term rehabilitation training to regain daily function [4]. However, traditional rehabilitation heavily relies on medical personnel, and the effectiveness of recovery is closely tied to the patient’s self-rehabilitation process. Without continuous monitoring or real-time feedback, rehabilitation outcomes may be suboptimal or even lead to deterioration of the condition [5].

Currently, major research directions focus on utilizing exoskeletons or imaging technology to aid rehabilitation. For example, exoskeletons can provide movement assistance and improve rehabilitation outcomes [6,7], while imaging technology can monitor patients’ progress through motion capture and data analysis [8,9]. However, these technologies have certain limitations. Exoskeletons may reduce patient acceptance due to discomfort, and their production and maintenance costs are often too high. Imaging technology requires high-performance equipment and professional operation, which limits its widespread application. Therefore, how to reduce costs while improving the usability and comfort of technology has become an important challenge.

The recent rise in edge computing has provided a promising solution for the rehabilitation field. Wearable devices integrated with edge computing can instantly collect and analyze patients’ limb movement data. It provides immediate feedback to determine whether the movements align with expectations [10]. Additionally, they can provide medical personnel with access to long-term records on the patient’s rehabilitation progress. However, edge computing still needs to overcome challenges such as sensor data accuracy, device computing power, and system usability in practical applications [11].

**Figure 1:**
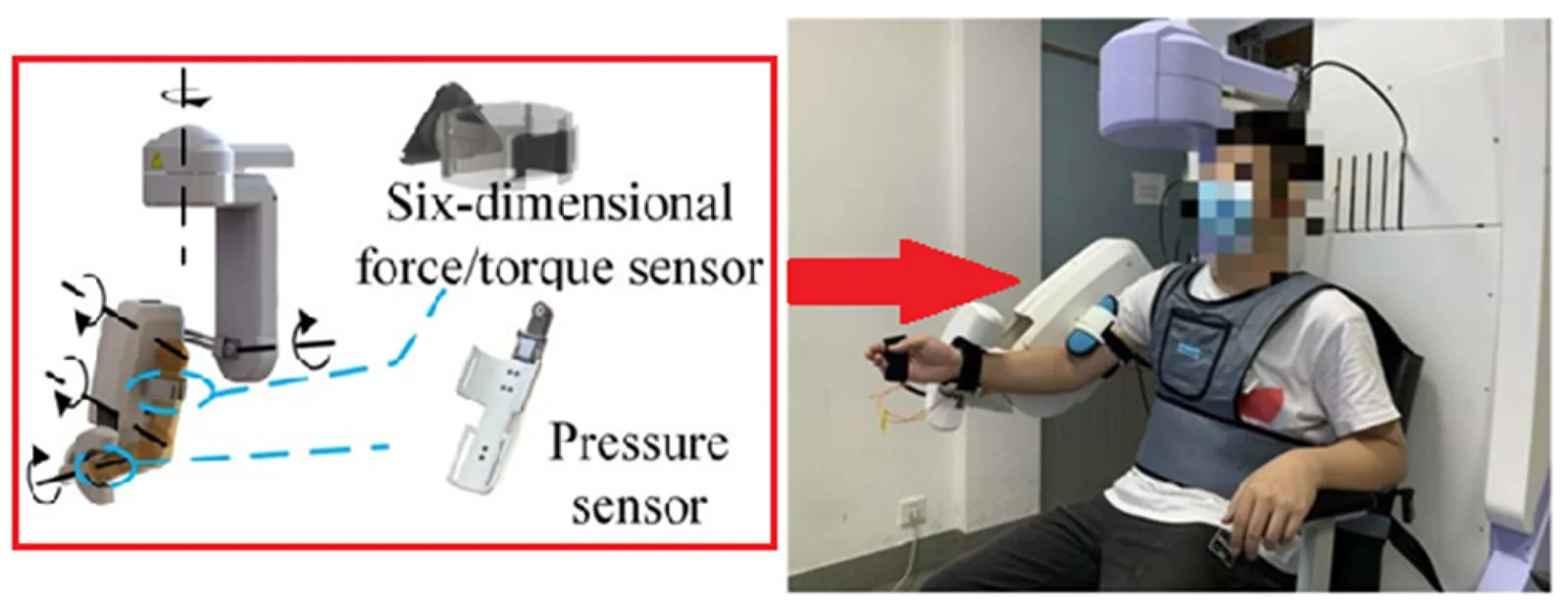
The mechanism structure of Armule (the black arrows indicate the rotational directions of the joints) [12].

In the field of rehabilitation, many articles have explored the application of various technologies. Chang He et al. proposed “Armule”, an upper-limb rehabilitation exoskeleton designed to imitate the natural limb movement based on “postural synergy” [12]. Unlike conventional rehabilitation devices that are often bulky and high-cost, Armule adopts a unique mechanical structure driven by two motors to control five joints, significantly reducing manufacturing costs and user burden while effectively reproducing the natural movement of the human arm, as shown in Figure 1. Their experimental results showed significant improvements in upper limb motor function, movement smoothness, and daily living abilities. This highlights the potential of “postural synergy”-based mechanical designs to enhance rehabilitation outcomes.

The application of imaging technology has also made significant progress in rehabilitation monitoring. Eojin Rho et al. proposed a hand rehabilitation system that combines visual detection [13]. This uses a deep learning framework called “DEEPOSE-Net” paired with an 8-degree-of-freedom soft robotic glove. By capturing the patient’s arm movements and interaction postures with objects via a camera, the system actively assists the patient in executing common daily movements such as grasping and pinching. Their experimental results showed superior immediacy and accuracy. This highlights the potential of combining visual assistance technology with robotic gloves to reduce patients’ reliance on expensive equipment or specialized operators.

Meanwhile, the rapid development of edge computing technology has provided new possibilities for rehabilitation applications. Zhihan Lv et al. developed a wearable rehabilitation system that integrates edge computing and cloud technology to implement real-time movement monitoring and local data processing [10]. Comparative evaluations revealed that offloading processing tasks to edge devices significantly optimizes diagnostic accuracy, transmission latency, and computational efficiency. These advancements validate the potential of edge computing in decentralized rehabilitation monitoring.

This article aims to design and implement an intelligent rehabilitation system based on edge computing to improve the accuracy and convenience of rehabilitation, thereby promoting rehabilitation treatment as a more efficient and customized process. Conceived as a home-based rehabilitation assistive tool rather than a certified medical device for clinical deployment, the primary objective is to establish a remote platform that potentially alleviates the commute burden on patients. To ensure the technical framework aligns with practical requirements, professional rehabilitation physicians from Chi Mei Medical Center and private clinics were consulted during the initial design phase to define standard exercise behaviors. By integrating an STM32 microcontroller with a wearable device, the system enables real-time monitoring and effective analysis of rehabilitation movements. To fully utilize the performance of the selected microcontroller, NanoEdge AI is employed as the artificial intelligence core for edge computing. To enhance user-friendliness, the STM32 communicates with a computer via Bluetooth. Historical rehabilitation data is accessed from a database to record the patient’s rehabilitation progress, providing a reliable analytical foundation for subsequent medical personnel. Furthermore, a custom website allows users to view historical data on mobile phones or computers, thereby streamlining the management of rehabilitation data for medical personnel and improving the efficiency and accuracy of overall treatment.

## 2. System Structure

**Figure 2:**
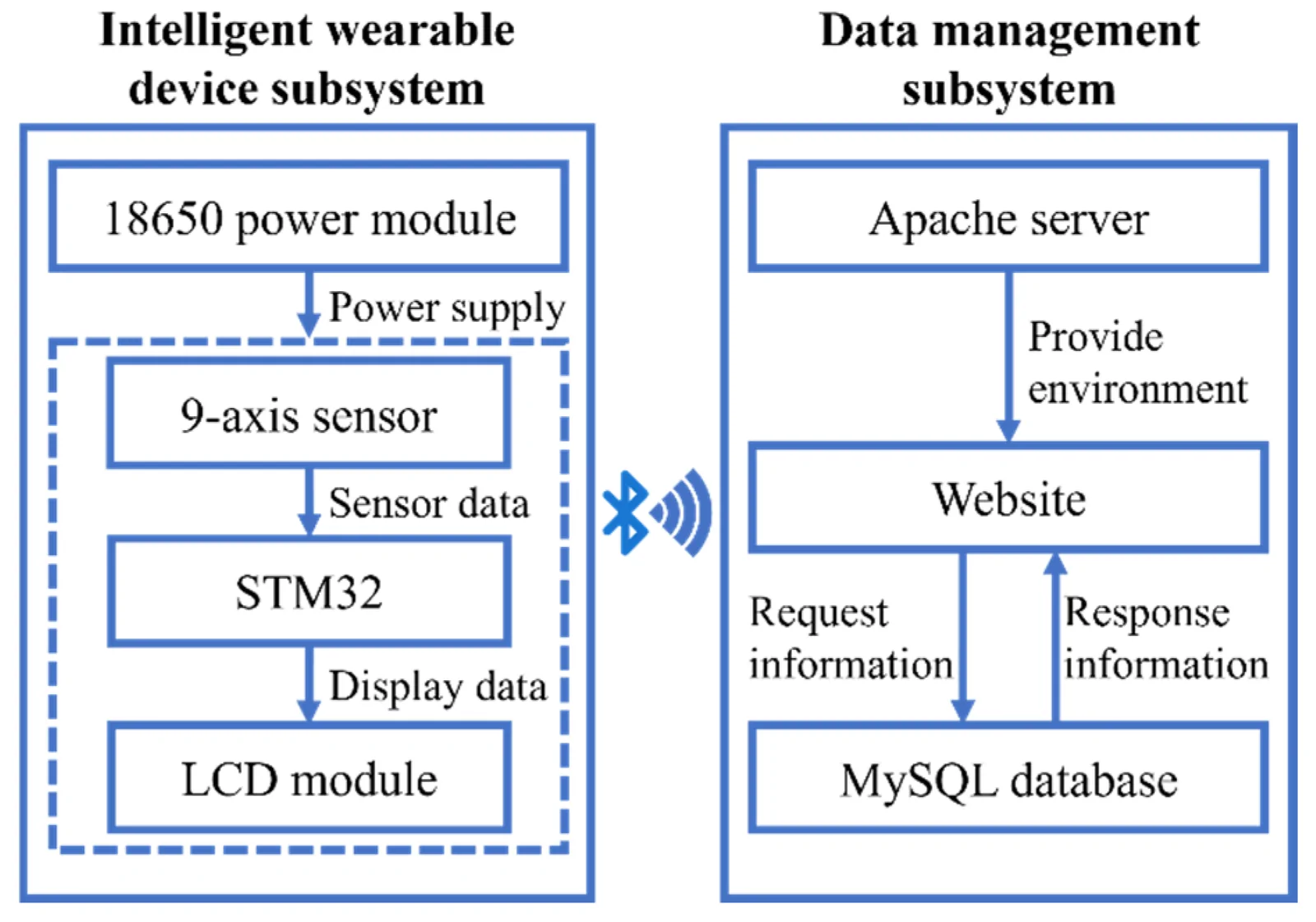
The system structure of the intelligent wearable rehabilitation system.

The system structure proposed in this article can be divided into two main subsystems: the intelligent wearable device subsystem and the data management subsystem. The intelligent wearable device subsystem is responsible for monitoring the patient’s rehabilitation movements and processing the motion data from the 9-axis sensor (TDK InvenSense, San Jose, CA, USA) through the STM32. The STM32 utilizes an STM32F103ZET6 microcontroller (STMicroelectronics, Geneva, Switzerland) as its core unit. Featuring an ARM Cortex-M3 core (ARM Holdings plc., Cambridge, UK) operating at a maximum frequency of 72 MHz, along with 512 KB of Flash memory and 64 KB of SRAM, the microcontroller provides sufficient computational capacity to handle real-time sensor data and edge AI inference efficiently. For hardware integration, the microcontroller features various communication interfaces, including UART, I^2^C, and SPI, which are utilized to connect with the sensor and Bluetooth modules. This hardware configuration ensures both system stability and power efficiency suitable for continuous wearable applications. After the data is classified and the accuracy is evaluated, it is transmitted to the data management subsystem through the Bluetooth module. The data management subsystem is built on the XAMPP platform, which includes the MySQL (Version 8.0) database and the Apache server (Version 2.4.58), for storing and managing data collected from the wearable device. Through PHP (Version 8.1.10) and JavaScript (Version ES14), a web interface is developed for patients and medical personnel to view rehabilitation progress and motion accuracy results in real time across different devices [14]. Figure 2 illustrates the interaction between the intelligent wearable device subsystem and the data management subsystem. Sensors collect movement data, and the microcontroller processes it before sending it to the server. The processed data is then stored and displayed for users to review.

### 2.1. Intelligent Wearable Device Subsystem

**Figure 3:**
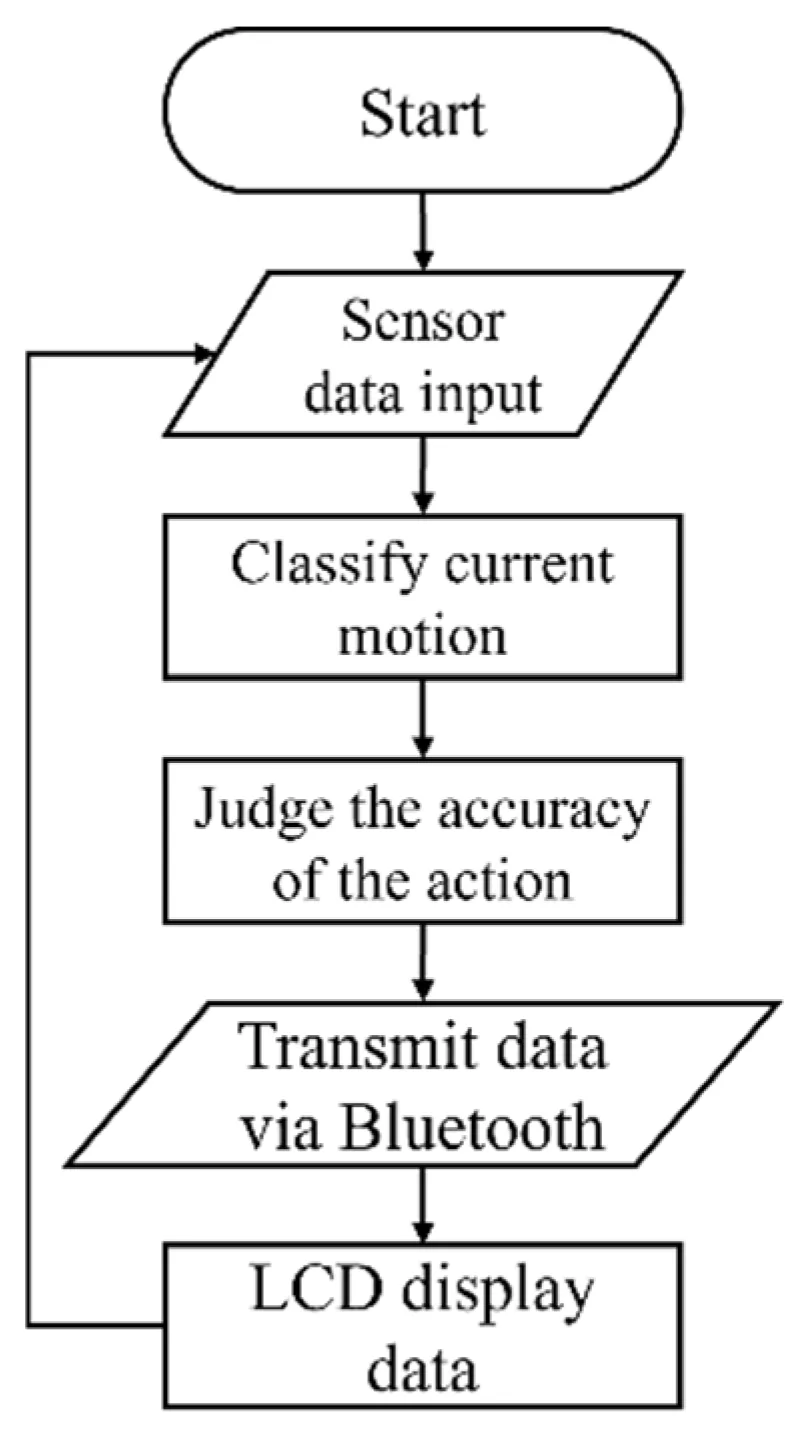
The flowchart of the intelligent wearable device subsystem.

Figure 3 illustrates the flowchart of this subsystem. When powered on, the sensors capture the patient’s motion data and transmit it to the STM32. The NanoEdge AI model classifies the current motion. It identifies the type of movement performed by the patient and further evaluates its accuracy. Once the assessment results are generated, the system transmits the data to the data management subsystem via Bluetooth. At the same time, the LCD module presents the relevant data on the screen. The user receives immediate motion feedback. This design improves convenience and accuracy of the rehabilitation process.

This subsystem requires a stable power source. This article uses 18650 lithium batteries as the main power source. These batteries are widely applied in electronic devices because of their high capacity, stability, and rechargeable nature [15,16]. The design allows the wearable device to operate for long periods. It also reduces the need for frequent battery replacement and improves overall convenience and reliability. The power expansion module provides stable 3.3 V and 5 V outputs. These voltages are suitable for the STM32 and peripheral modules, including the 9-axis sensor, Bluetooth module, and LCD module.

**Figure 4:**
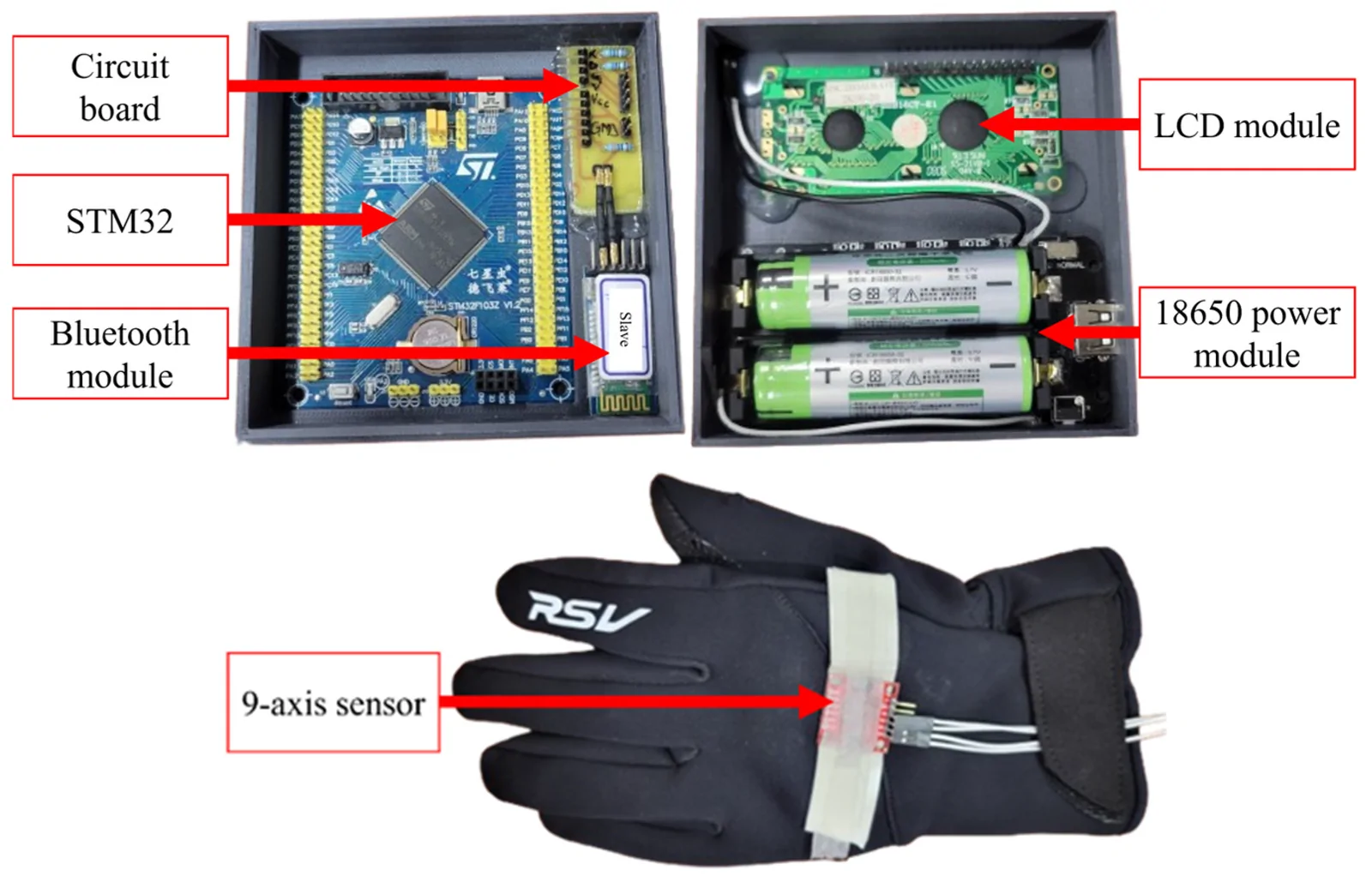
Hardware configuration of the intelligent wearable device.

To ensure structural integrity and visual quality, the outer shell was designed in-house. It was manufactured with 3D printing technology according to the required size and function. The shell protects the internal electronic components. It also improves portability and comfort, making the device more suitable for wearable applications. The sensor components are connected through pre-drilled holes in the shell. Their wires are fixed on the glove surface. This design captures subtle wrist movements more accurately. It increases the precision of posture recognition and enhances the stability of the entire system. Figure 4 illustrates the physical appearance of the intelligent wearable device subsystem.

#### 2.1.1. 9-Axis Sensor and Communication Protocol

The 9-axis sensor uses I^2^C as its default communication interface. The voltage level of the AD0 pin determines the slave address. When the AD0 is connected to a low potential, the I^2^C address is 0x68. When it is connected to a high potential, the address becomes 0x69. This design allows the flexible configuration of the same or different sensor models on a single I^2^C bus. It prevents address conflicts and improves system integration.

**Figure 5:**
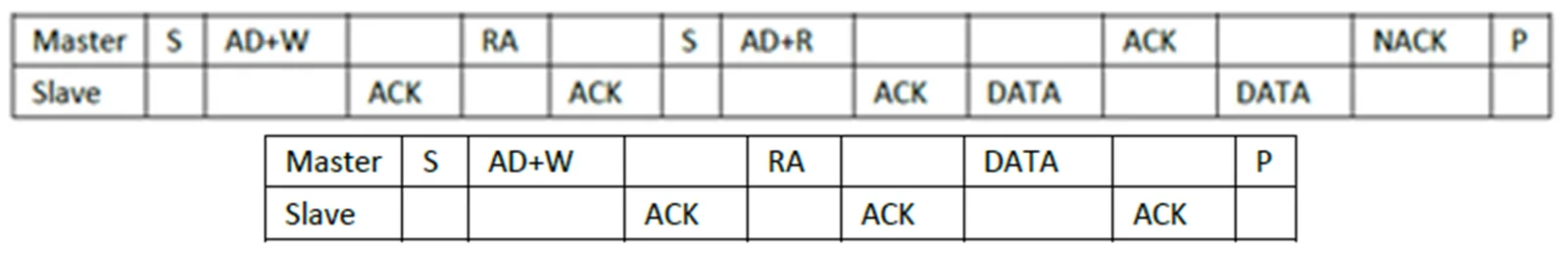
The 9-axis sensor uses the I^2^C communication protocol, with the top for reading and the bottom for writing.

During I^2^C communication, data transmission between the master and the slave follows a defined communication protocol. The protocol ensures data integrity and transmission accuracy. Figure 5 illustrates an example of data transmission.

**Figure 6:**
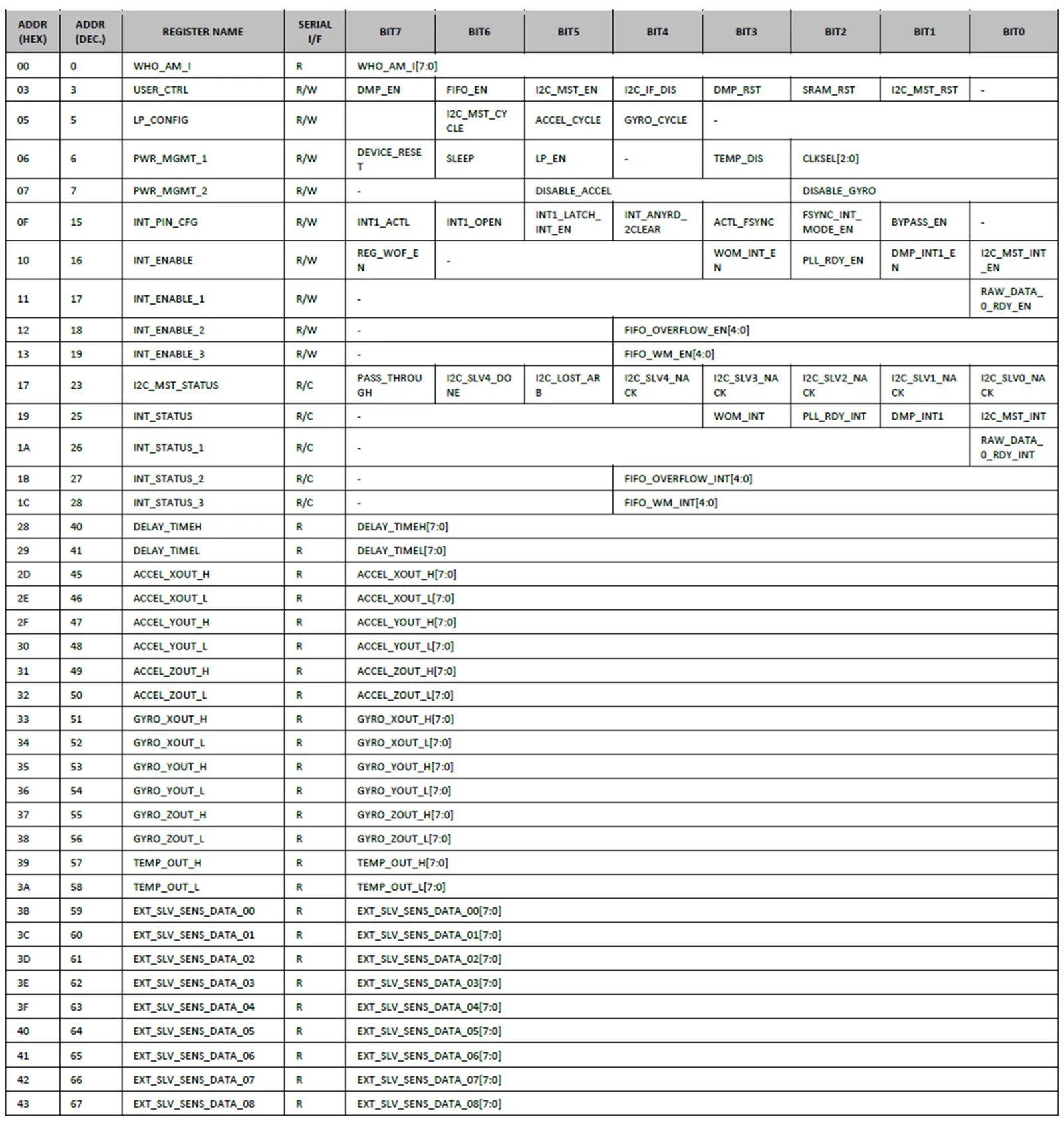
The Bank 0 register address provided in the 9-axis sensor specification sheet.

The 9-axis sensor contains four internal register banks. Each bank has its own independent register set. This multi-bank structure allows flexible configuration of parameters according to different application needs. It improves the accuracy and stability of data processing. The modular and highly integrated design helps the intelligent wearable device provide reliable and consistent data during real-time motion recognition and posture analysis. This design also enhances the user’s interaction experience and overall application performance. Figure 6 illustrates the register functions of Bank 0 in the 9-axis sensor.

#### 2.1.2. Posture Analysis

The 9-axis sensor provides data from a three-axis gyroscope, a three-axis accelerometer, and a three-axis magnetometer. These data correspond to the object’s angular velocity, linear acceleration, and geomagnetic direction. To analyze the rotation state of an object in space, the original 9-axis data are not sufficient to describe its attitude. The data must be converted into meaningful angular information, such as Euler angles. This conversion is performed through attitude analysis algorithms [17].

**Figure 7:**
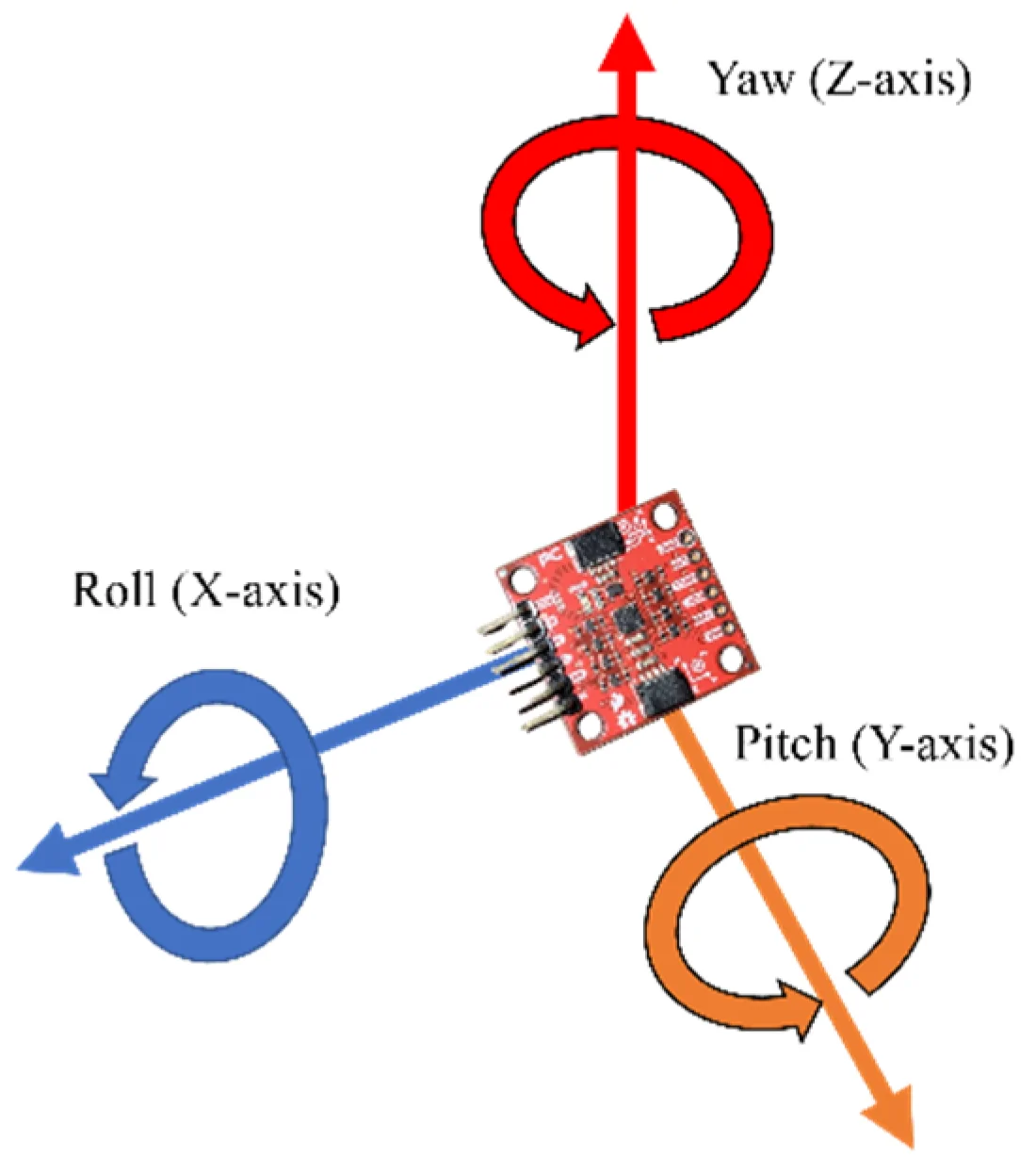
Euler angles diagram (the arrows indicate the positive rotational directions defined by the right-hand rule).

Euler angles describe the rotation of a three-dimensional object in space. They are defined by three parameters: roll, pitch, and yaw. However, Euler angles can cause a gimbal lock problem when certain rotation angles occur. This problem often appears when the pitch angle approaches ±90 degrees. At that point, one degree of freedom is lost, and part of the rotational information disappears. The result is reduced continuity and lower accuracy in attitude representation [18]. Figure 7 illustrates the relationship between three-axis angular velocity and Euler angles.

To address the gimbal lock, quaternions are often used as an alternative. The quaternion is a mathematical representation that maps rotation information from four-dimensional space to three-dimensional space. It can describe the rotation of a three-dimensional object without losing any degrees of freedom [19]. Using quaternions in rotation calculations improves the stability of attitude estimation. It also increases computational efficiency and enhances the accuracy of applications such as motion classification, robot control, and virtual reality.

During attitude analysis, the angular velocity data are used in the quaternion differential equation, as in (1). The equation is solved to obtain the rate of change in the quaternion. A numerical integration method then updates the previous quaternion with the new value, as in (2). The result provides the current quaternion that represents the object’s attitude at that moment [20].

(1) $$\dot{q}=\frac{1}{2}q⊗\omega_{q}$$

$\dot{q}$: The rate of attitude change over time.

q: The quaternion of the current posture, expanded as $\left[\begin{array}{c} q_{0}\\ \begin{array}{c} q_{1}\\ q_{2}\\ q_{3} \end{array} \end{array}\right]$.

$\omega_{q}$: The angular velocity vector measured by the gyroscope, expanded as $\left[\begin{array}{c} 0\\ \begin{array}{c} g_{x}\\ g_{y}\\ g_{z} \end{array} \end{array}\right]$.

(2) $$q_{new}=q+\dot{q}\cdot \Delta t$$

$q_{new}$: The new attitude quaternion is calculated.

∆t: Time interval.

In theory, continuous attitude estimation can rely on the angular velocity data from the gyroscope. However, the gyroscope has offset errors and noise. Long-term integration of these errors causes serious drift in the attitude estimation results and reduces the overall system accuracy. To suppress such accumulated errors, other sensors are required for correction. The accelerometer measures the direction of Earth’s gravity and provides an attitude reference when the object is stationary. The magnetometer measures the direction of Earth’s magnetic field and provides a reference for heading correction. By fusing the data from the gyroscope, accelerometer, and magnetometer, the weaknesses of each sensor can be compensated. This approach improves both the accuracy and stability of attitude estimation [21].

The Madgwick filter reduces the difference between the observed and estimated vectors through gradient descent, as in (3). It derives a gradient vector, expressed as s. This vector is fused into the quaternion differential equation using a scale factor *β*. This process reduces the influence of individual sensor errors and provides a stable estimation of the final Euler azimuth angle. It also improves the reliability and practicality of the system.

(3) $$\dot{q}=\frac{1}{2}q⊗\omega_{q}-\beta \cdot s$$

β: Algorithm gain.

s: The correction direction calculated using gradient descent, expanded as $\left[\begin{array}{c} \begin{array}{c} s_{0}\\ s_{1} \end{array}\\ s_{2}\\ s_{3} \end{array}\right]$.

After the quaternion calculation and update are completed, the result is converted into Euler angles, which serve as the final basis for judging the attitude change in edge computing.

Quaternion to Roll conversion, as in (4) [22].

(4) $$Roll=\tan^{-1}2(\frac{2\left(q_{0}q_{1}+q_{2}q_{3}\right)}{1-2(q_{1}^{2}+q_{2}^{2})})$$

Quaternion to Pitch conversion, as in (5) [22].

(5) $$Pitch=\sin^{-1}\left(2(q_{0}q_{2}-{\;q}_{3}q_{1})\right)$$

Quaternion to Yaw conversion, as in (6) [22].

(6) $$Yaw=\tan^{-1}2(\frac{2\left(q_{0}q_{3}+q_{1}q_{2}\right)}{1-2(q_{2}^{2}+q_{3}^{2})})$$

#### 2.1.3. Wireless Transmission System

This system uses the HM-10 Bluetooth module as the core of wireless communication. The Bluetooth module supports the Bluetooth 5.0 low energy standard. Compared to traditional Bluetooth 2.0, BLE features drastically reduced power consumption and faster connection speeds, making it highly suitable for continuous short-range data transmission in wearable applications. These advantages make it more practical than other common wireless technologies [23]. Table 1 compares the characteristics of Bluetooth with those of other wireless communication technologies. The rehabilitation scenario requires low power consumption, short-range transmission, and reliable signal performance. Compared to Wi-Fi, Zigbee, and LoRa, Bluetooth offers more stable communication over short distances and better power management.

The HM-10 module supports AT commands for configuring master or slave operation and adjusting the UART baud rate to match the STM32, ensuring reliable serial communication. To improve power efficiency, the system configures a fixed peer MAC address and exchanges data through predefined BLE GATT service and characteristic UUIDs. This targeted addressing mechanism enables rapid reconnection to the intended device while reducing repeated device discovery, scanning, and advertising during normal operation. As a result, the proposed approach provides reliable, low-latency, and energy-efficient wireless data transmission between the wearable device and the receiver.

### 2.2. Data Management Subsystem

**Figure 8:**
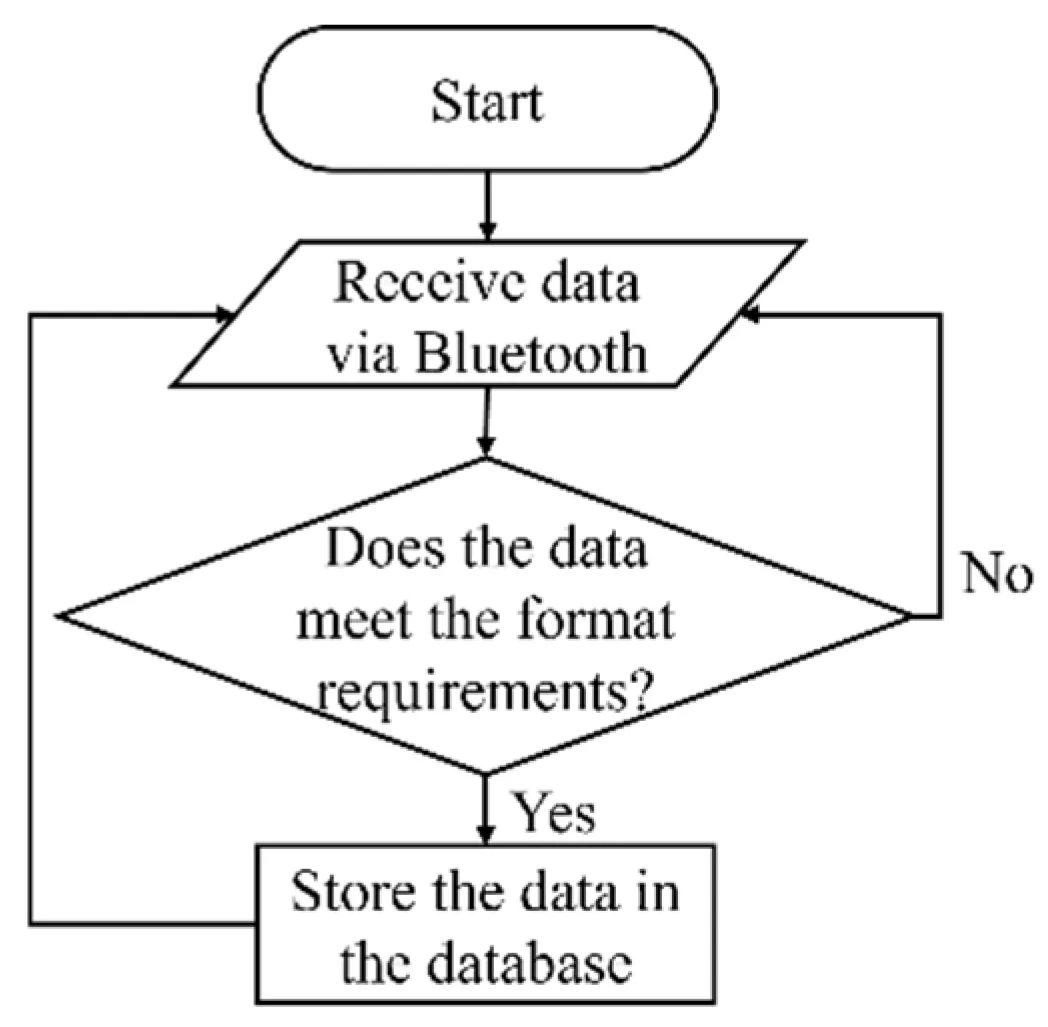
The flowchart of the data management subsystem.

The data management subsystem receives, processes, and stores data transmitted from the intelligent wearable device subsystem through the Bluetooth module. It ensures the integrity and traceability of rehabilitation data. The received data are first checked for format compliance. If the data do not meet the required format, they are discarded. The system then waits for new data transmission. This process ensures that the data stored in the database is accurate and meaningful. Once the data passes the format verification, Python (Version 3.11) stores the processed data in a MySQL database. The database maintains a complete record of the patient’s rehabilitation history. Figure 8 illustrates the data processing flowchart of the system after receiving the data.

#### 2.2.1. Encrypted Login

**Figure 9:**
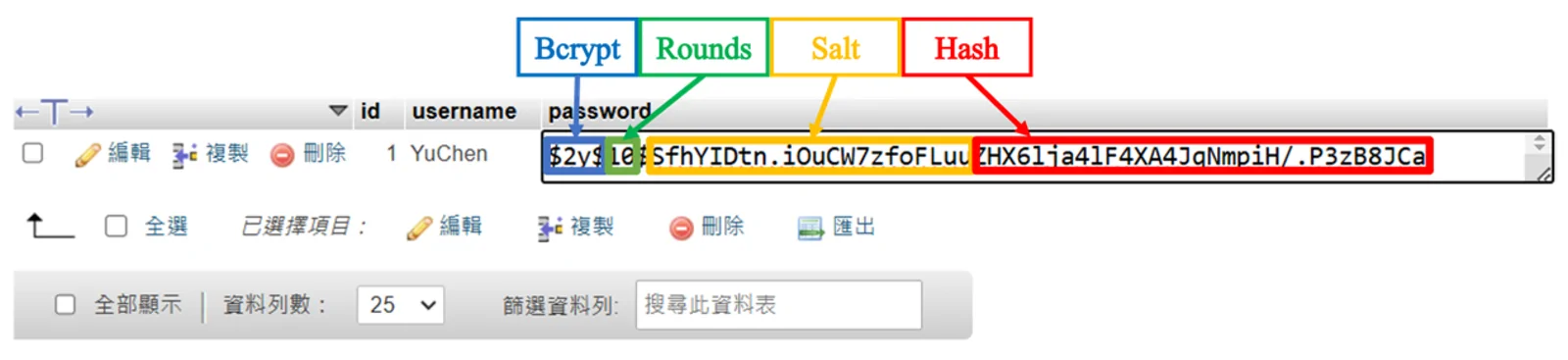
Encrypted password in the database.

To ensure user privacy and data security, this system uses the Bcrypt hashing algorithm to store user passwords. Bcrypt improves password protection through internal salting and key stretching techniques [24]. The salting process generates a different hash value even when the same password is used. Key stretching increases the difficulty of brute-force attacks by computing the hash hundreds to thousands of times. Figure 9 illustrates the database structure after the password is encrypted using Bcrypt. This structure prevents rainbow table attacks and brute-force attacks. It also ensures strong security for the password information stored in the database.

1. Bcrypt: Indicates that Bcrypt encryption is used.
2. Round: Represents the strength of the hash operation. A higher value results in a long computation time and increases the difficulty of cracking.
3. Salt: Used to ensure that the same password produces different hash results each time.
4. Hash: The encrypted result is calculated based on the user-input password and the salt value.

#### 2.2.2. Database Management

This article uses XAMPP (Version 7.4.33) to build a local server environment. The database system and web interface are created through its integrated MariaDB. The web interface presents the rehabilitation data in real-time, and medical personnel can monitor the patient’s home rehabilitation progress. This design enables providers to offer more accurate diagnoses and individualized recommendations based on rehabilitation status.

**Figure 10:**
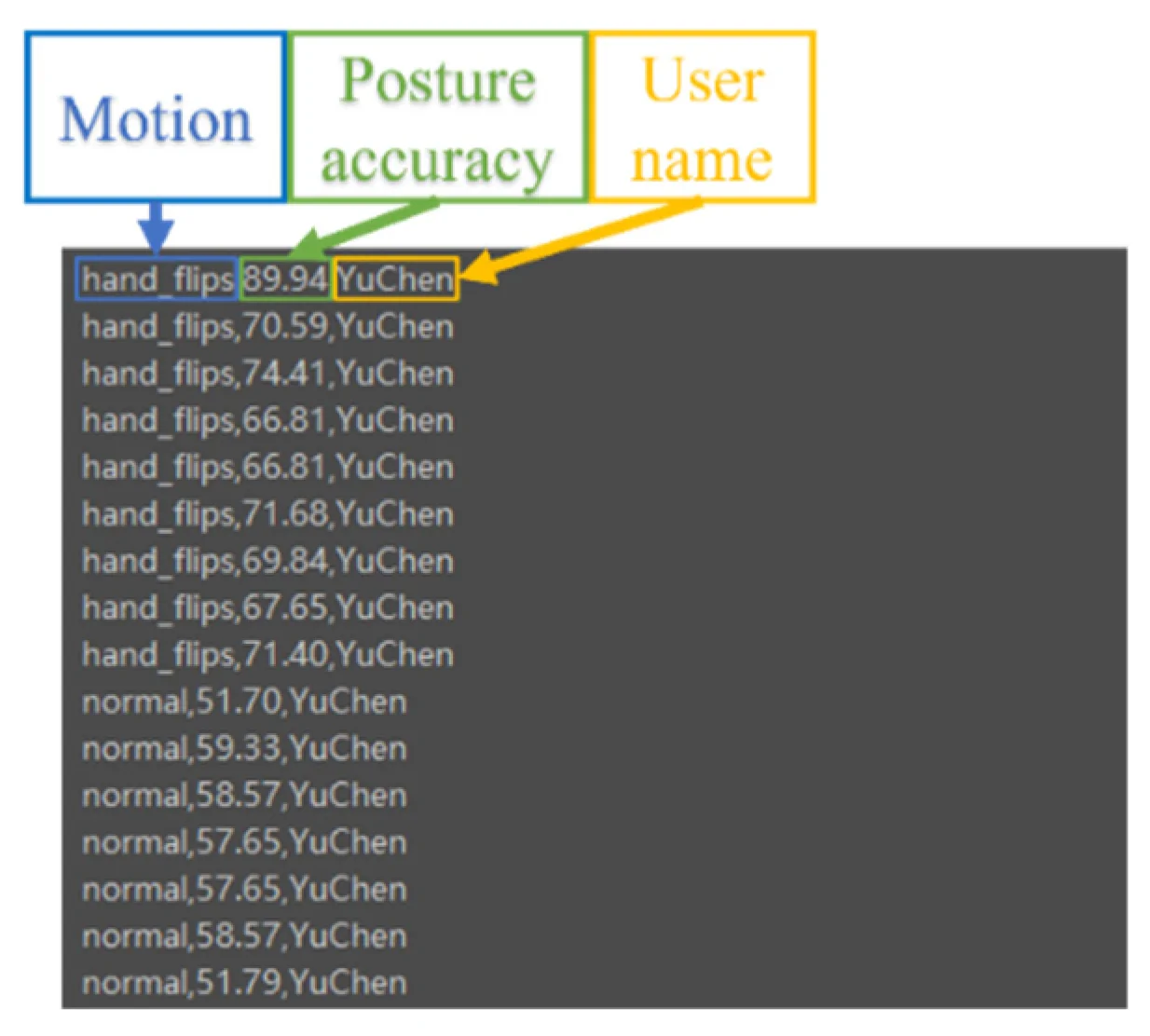
The correct format is read by the receiving end.

After the intelligent wearable device subsystem completes motion recognition, the data are sent to the computer through Bluetooth. A Python program performs real-time format validation on the received data. Each data record must contain three fields: motion type, posture accuracy, and user name. After validation, the system checks whether the user name exists in the database. The data are written into the database only when the user is valid. Figure 10 illustrates the data format obtained through the UART serial interface.

#### 2.2.3. Website

**Figure 11:**
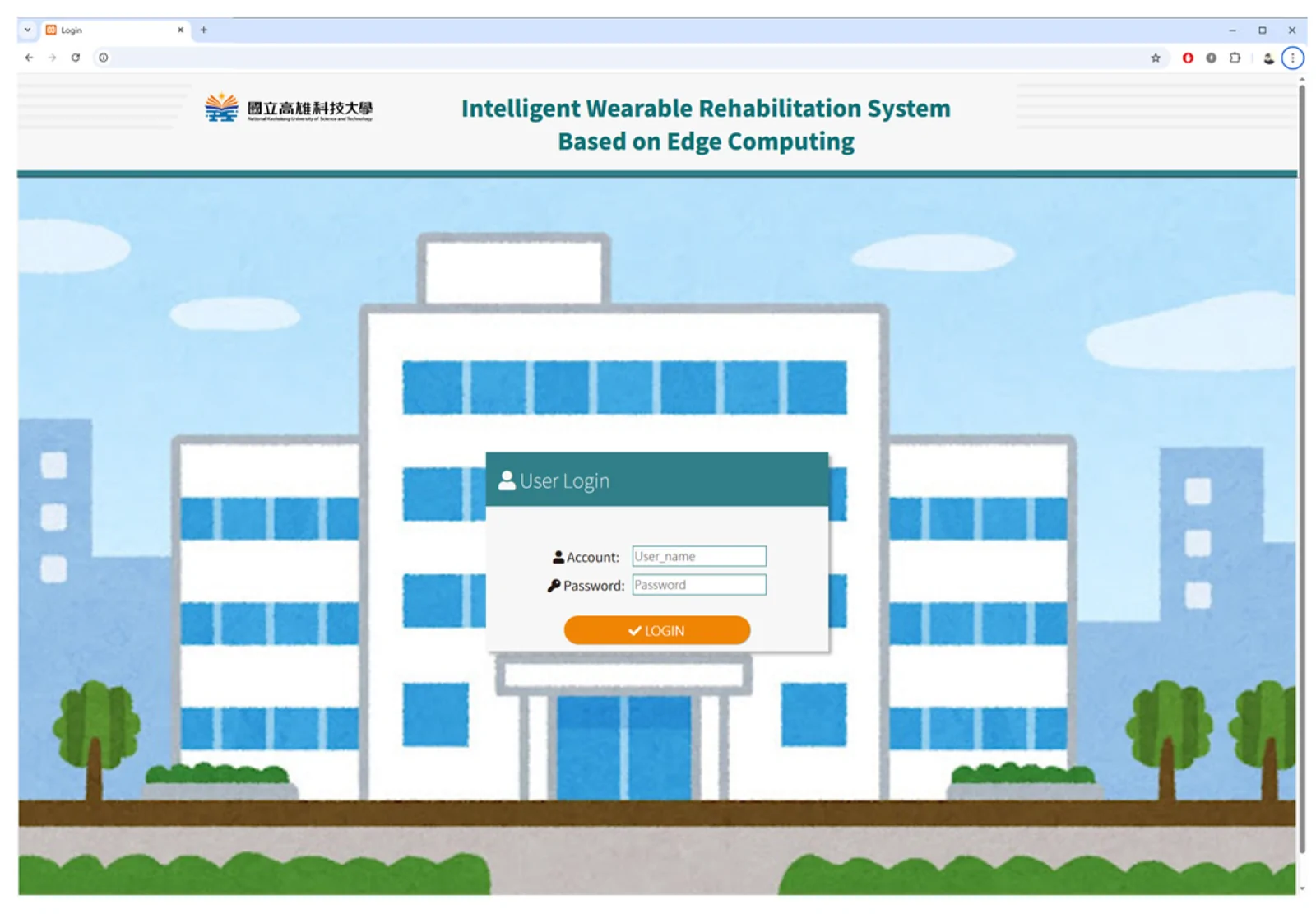
Login interface of the website.

This system presents a self-designed web interface that enables operators to monitor past rehabilitation records. The login system uses the session_start () function to manage user session states. This mechanism prevents unauthorized users from accessing internal pages by entering a URL directly. If an unauthorized user attempts to open an internal page, the system detects the invalid session and redirects the user to the login page. Figure 11 illustrates the self-designed login interface.

**Figure 12:**
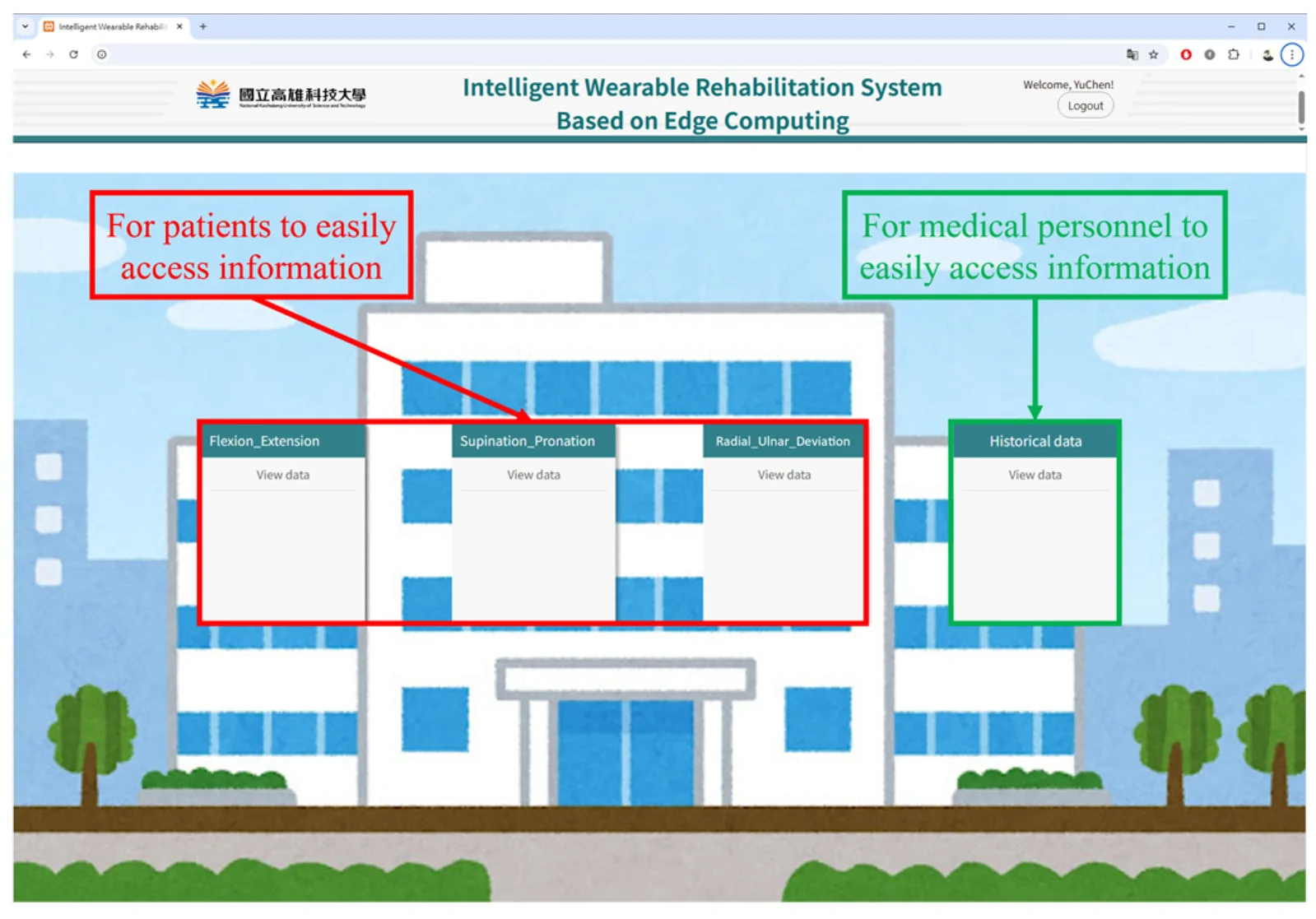
Homepage of the website.

After a successful login, the system homepage displays four options. The three options on the left are designed for patients. They allow patients to view their personal rehabilitation data. The option on the right is designed for medical personnel. It provides full access to the database. Figure 12 illustrates the homepage displayed after login.

**Figure 13:**
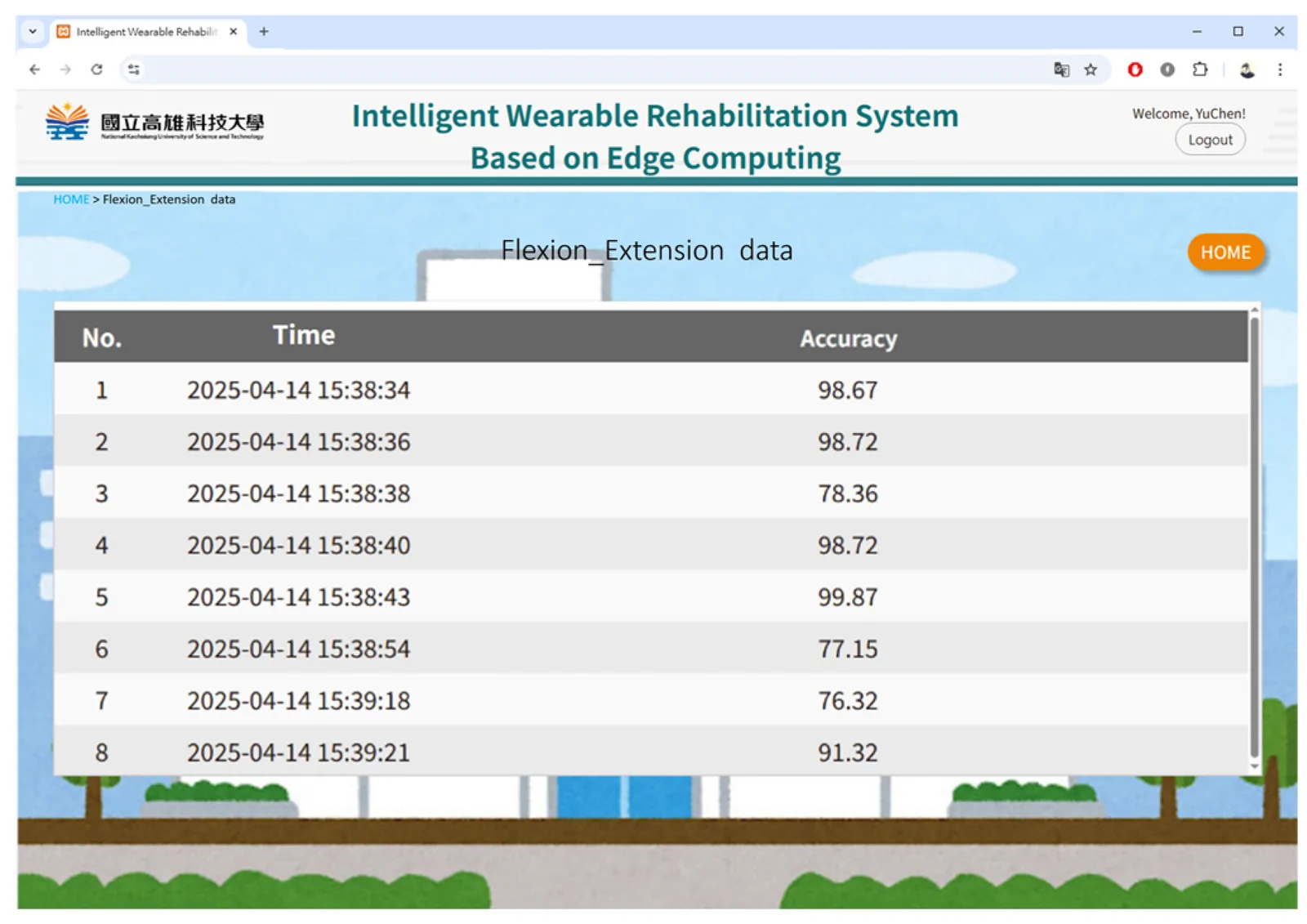
The page for Flexion_Extension.

Patients can view their historical records for each motion by clicking on the “View data”. For example, they can check the performance of “Flexion_Extension”. The screen lists all records in chronological order and shows the time of the performance and the accuracy percentage. This information helps patients understand their progress and track their rehabilitation results over time. Figure 13 illustrates the page for “Flexion_Extension”.

**Figure 14:**
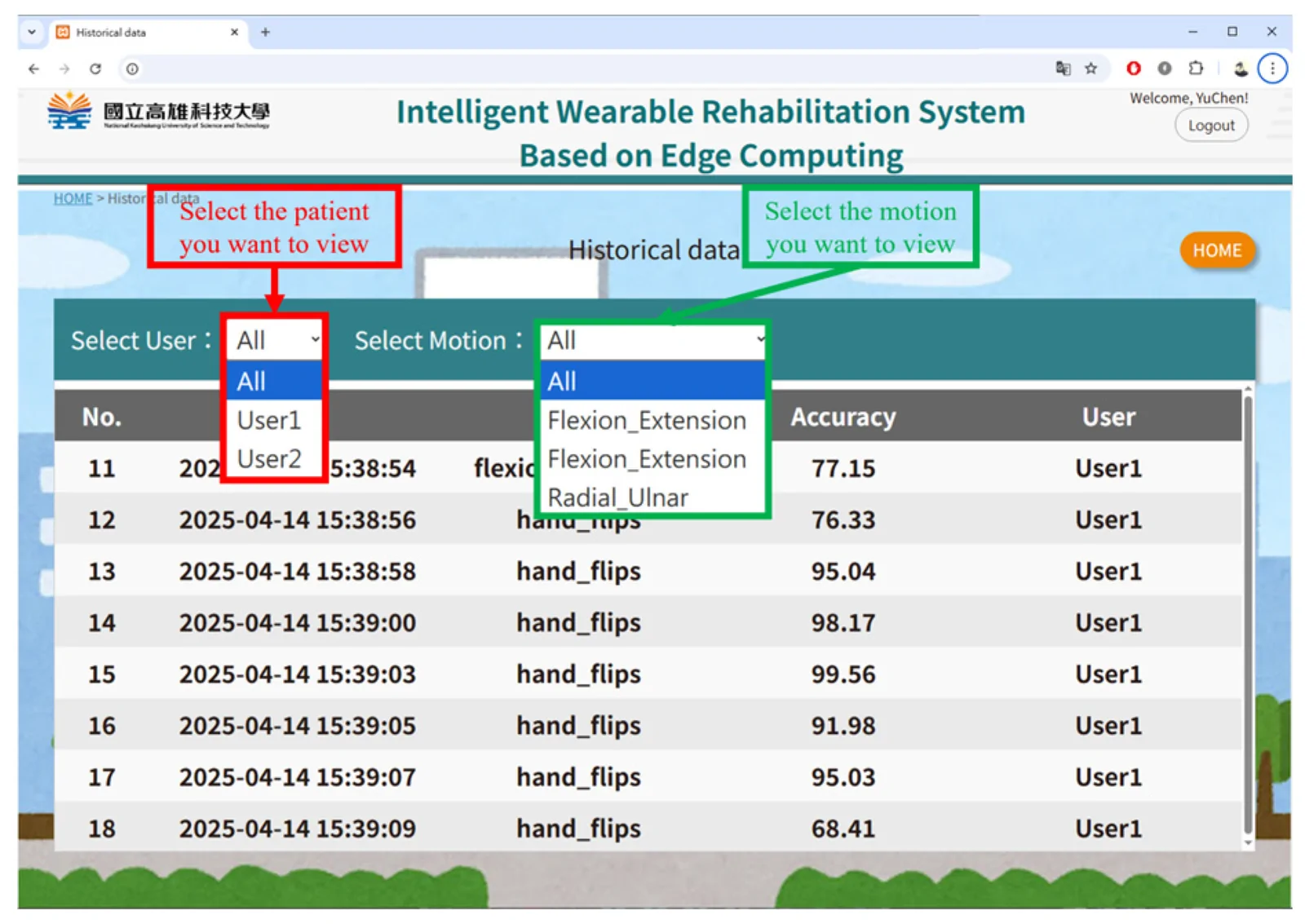
The user interface for medical personnel.

The historical data option is designed for medical personnel. It provides full access to the database. The interface includes drop-down menus that allow users to filter specific patients or specific motion data. This design enables medical personnel to review long-term rehabilitation records efficiently. It also improves the efficiency of clinical decision-making and treatment support. Figure 14 illustrates the user interface for medical personnel.

### 2.3. Model Dataset and Training

This section details the methodologies applied to build the specific rehabilitation dataset and the strategy for selecting an optimized lightweight model for edge inference.

#### 2.3.1. Model Training and Selection Strategy

To select the most suitable combination of data processing flow and classification model for real-time motion recognition, this article uses NanoEdge AI Studio (Version 4.6.0). The sensed data are first uploaded to the NanoEdge AI cloud platform, where it automatically explores combinations of data preprocessing and classifiers. The platform evaluates each combination based on the training results and recommends the optimal configuration while ensuring its memory footprint remains within the user-defined RAM and Flash limits of the target microcontroller. The NanoEdge AI studio operates by benchmarking and generating a specialized static library tailored specifically to comply with the hardware resource constraints of the target microcontroller. The generated static library, utilizing the selected Random Forest algorithm, is explicitly tailored to the target STM32 microcontroller rather than acting as a large-scale PC-level model, thereby rendering secondary optimization pipelines such as post-training pruning or quantization completely unnecessary.

**Figure 15:**
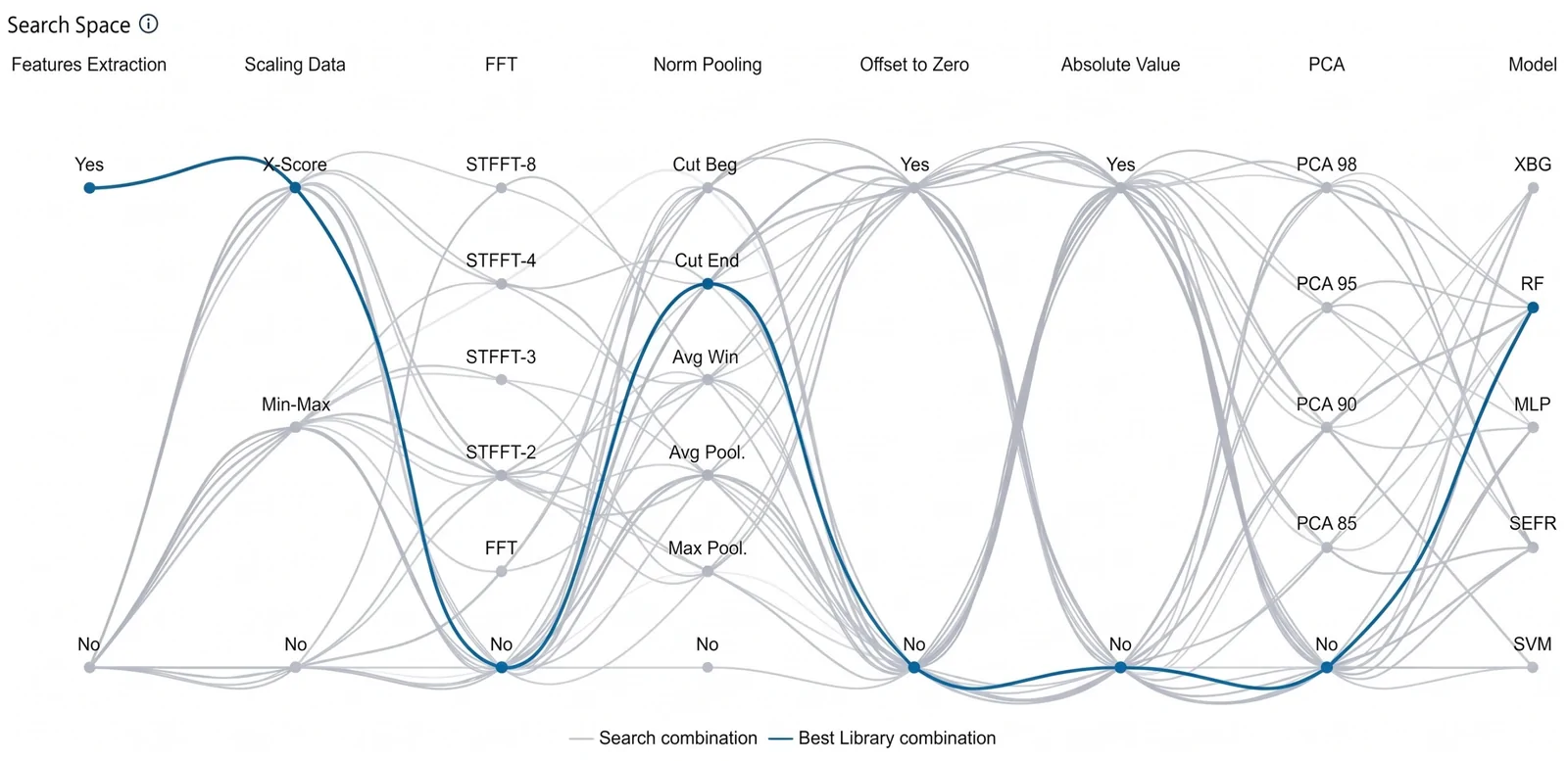
Parallel coordinates plot generated by NanoEdge AI.

Next, a parallel coordinates plot is used to explore optimization strategies [25]. Figure 15 illustrates the plot generated by NanoEdge AI. The plot visualizes the relationship between different parameter settings and overall performance. It clearly presents the effect of each parameter combination on model performance in the process from data preprocessing to classifier training.

Each curve in Figure 15 represents a complete combination of data processing flow and model configuration. The color of the line indicates the final performance. A darker color represents better model performance. To provide technical insight into the generated NanoEdge framework, the selected optimal pipeline implements the following operations:

1. Z-score normalization is applied to standardize the amplitude variations in the raw Euler angles, mitigating differences in execution speed or range of motion among different users.
2. Fast Fourier Transform is disabled. For these specific, slow-paced rehabilitation gestures, the raw temporal sequence of the spatial angles captures the kinematic motion better than frequency-domain features.
3. Cut End mode is adopted for data segmentation, which handles variable-length motion sequences by systematically aligning the data from the start of the gesture.
4. Principal Component Analysis is disabled to preserve the explicit physical meaning of the 3-dimensional spatial angles (roll, pitch, yaw) without information loss.

Regarding the model selection, the primary advantage of the Random Forest model is that its inference process relies on highly efficient decision-tree conditional branching. This results in incredibly low computational complexity and an exceptionally small memory footprint, which is perfectly suited for the limited RAM and Flash resources of the STM32F103ZET6 microcontroller.

#### 2.3.2. Dataset Construction

The physician personally wore the prototype rehabilitation glove to perform and record the specific rehabilitation motion data. This data was utilized for model development, where NanoEdge AI Studio automatically partitioned it into training and validation sets. The authors performed the same rehabilitation motions to construct an independent testing set. Each motion dataset was recorded over a continuous duration of approximately 1 min, generating 1800 to 2000 data points. Each data point explicitly captures the Euler angles corresponding to continuous motion instances within that timeframe. The data were imported into NanoEdge AI Studio in CSV format for preprocessing and training. After the system processes the input data, the platform generates visualizations that show the mean and standard deviation of each axis. They help us understand the differences between datasets at the data level.

**Figure 16:**
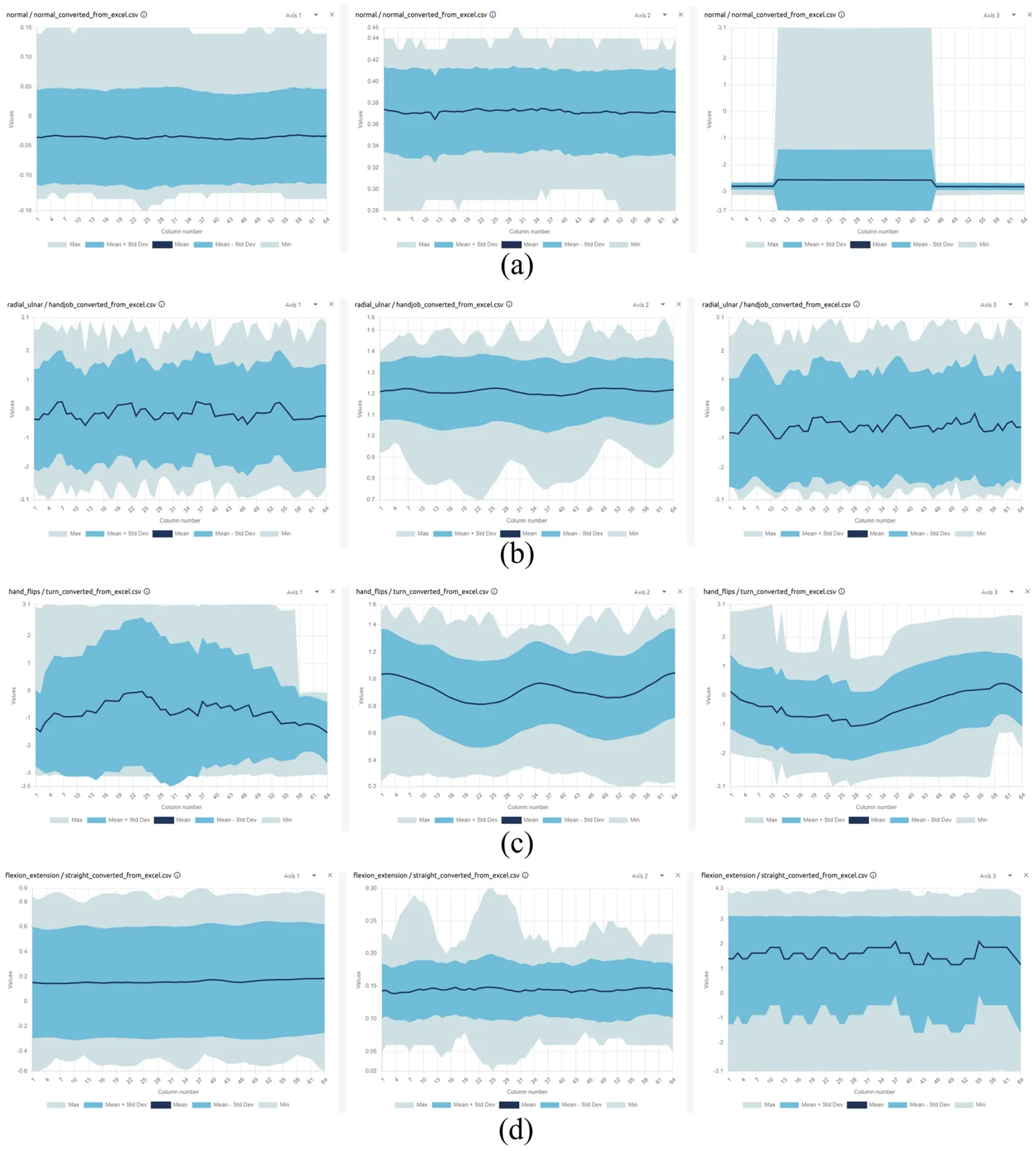
The dataset of each motion: (**a**) Static; (**b**) Radial_Ulnar_Deviation; (**c**) Pronation_Supination; (**d**) Flexion_Extension.

Figure 16 illustrates the data distribution of the “Static”, wrist radial and ulnar deviation (Radial_Ulnar_Deviation), wrist pronation and supination (Pronation_Supination), and wrist flexion and extension (Flexion_Extension). The horizontal axis represents the order of the input data. The vertical axis represents the sensed values. The three charts appear from left to right as roll, pitch, and yaw.

1. “Static” shows stable variation across the three axes. The Roll and Pitch axes have minimal standard deviations. This result indicates high stability and consistency during execution. It also shows that the user maintains reasonable posture control in a static state. The “Static” data serve as an important reference for identifying a static condition.
2. “Radial_Ulnar_Deviation” shows a large variation on the Roll and Yaw axes. These variations indicate lateral tilt wrist, which is a key feature of this movement. The movement can be identified by its high variability across multiple axes.
3. “Pronation_Supination” shows a large change on the Pitch axis. It is the only movement among the four with this pattern. The variation indicates forearm rotation along its axis. This feature provides an important basis for identifying this movement during classification and evaluation.
4. “Flexion_Extension” shows a large variation on the Yaw axis. This variation indicates that the movement relates to forward and backward wrist flexion. The movement also shows a stable trajectory. This stability enables the system to identify and classify the motion accurately.

## 3. Results

This section evaluates the stability and feasibility of the proposed intelligent rehabilitation system. It verifies the system’s effectiveness through practical operation and data observation. The experiment examines the system’s performance under different movements. It also explores the relationship between the monitored rehabilitation movements and the actual rehabilitation records. These results demonstrate the value of movement data as a basis for medical judgment.

### 3.1. Intelligent Wearable Device

**Figure 17:**
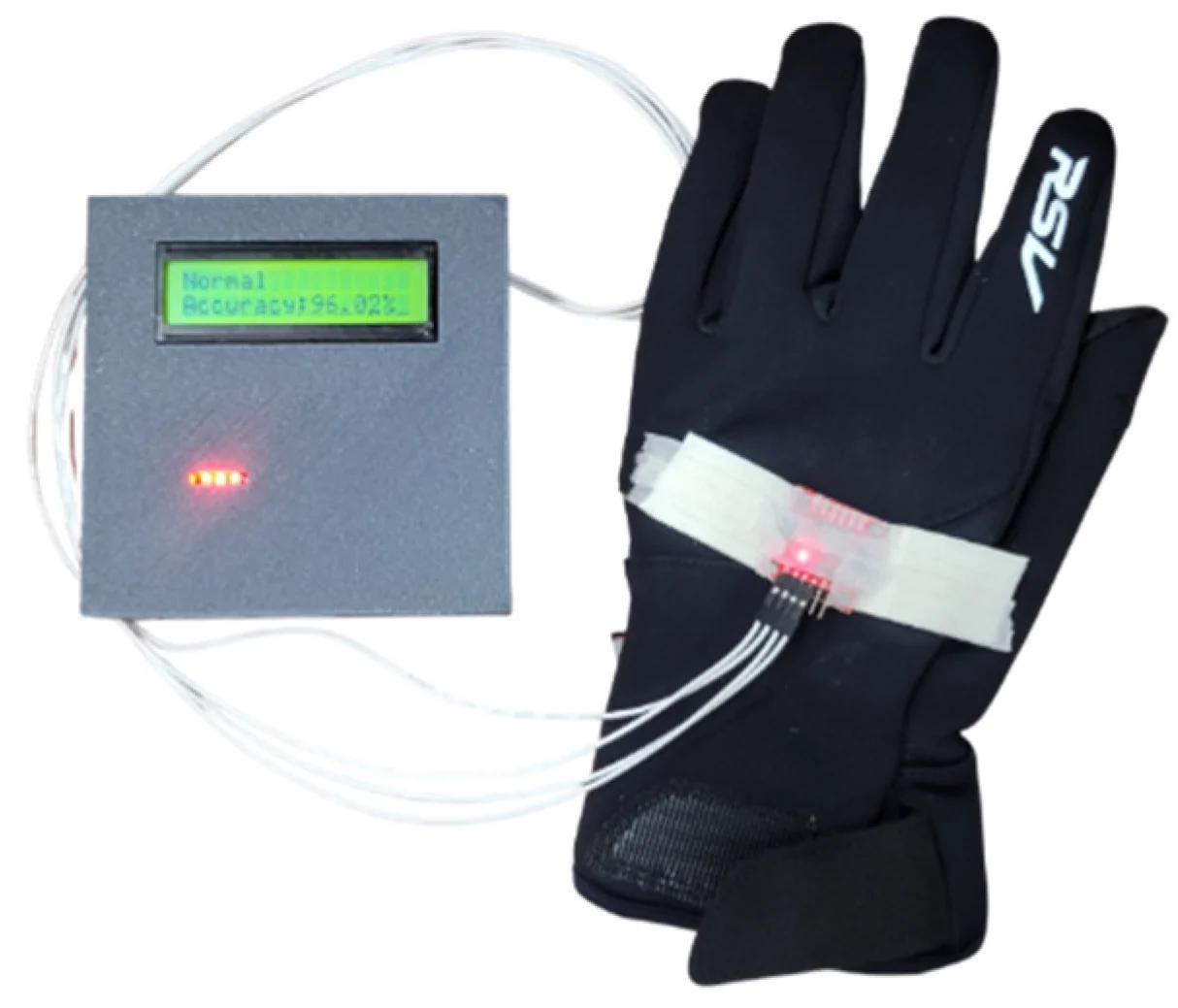
The physical device of the intelligent wearable system.

Figure 17 illustrates the physical device of the intelligent wearable device. The left side is the system control unit. It includes an LCD module and an LED power indicator. The right side is the wearable glove. It contains a sensing module that measures the user’s motion data in real-time. The glove is connected to the control unit via wires to provide real-time feedback and judgment functions.

### 3.2. Training Results

**Figure 18:**
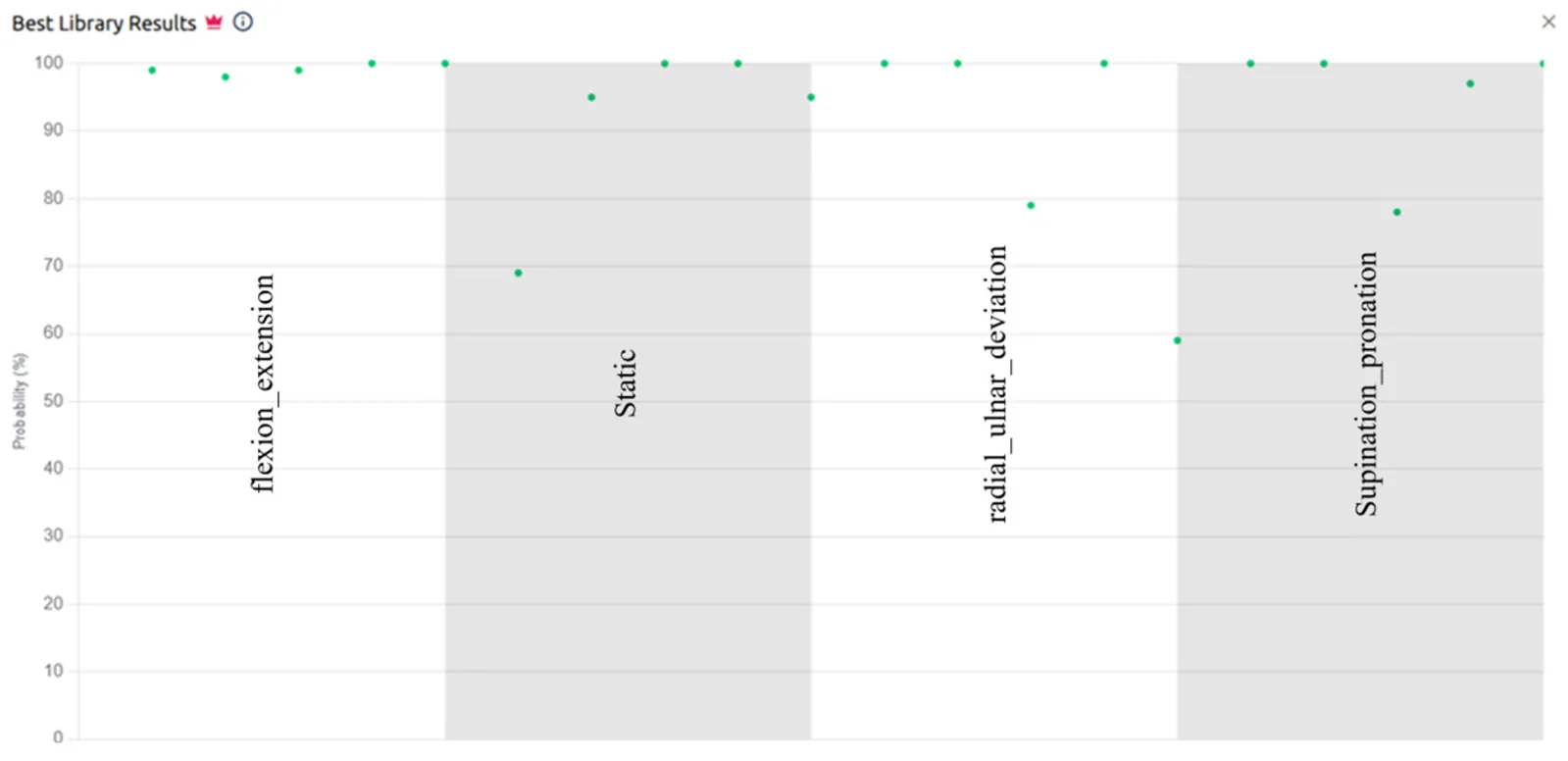
The results of motion classification (The red crown symbol denotes the theoretically best model).

Figure 18 illustrates the classification results of the best model generated using NanoEdge AI Studio. The horizontal axis lists the names of the motion categories. The four categories are Flexion_Extension, Static, Radial_Ulnar_Deviation, and Pronation_Supination. All data comes from the CSV training set imported into the system. The vertical axis shows the predicted probability for each motion category, which quantitatively measures the deviation between the user’s real-time Euler angles and the standard physician baseline.

Each green dot represents a prediction result from a test sample. It is important to distinguish between the model’s real-time postural feedback and its overall classification performance. The “probability” values shown in Figure 18 represent the model’s confidence scores. These scores are designed to be utilized directly as real-time visual feedback for the user to self-adjust their posture during prototype operation. Regarding the quantitative evaluation metrics for motion recognition, we employed an “Expert Baseline Comparison” methodology. Specifically, the training and validation sets were constructed using data recorded by a professional rehabilitation physician performing the standardized gestures. The authors performed the rehabilitation motions to serve as an independent test set. Quantitatively, our preliminary testing achieved a motion recognition accuracy exceeding 80%. Achieving this level on the edge successfully validates the technical feasibility of providing low-cost, low-latency, and privacy-preserving real-time feedback for home-based rehabilitation.

### 3.3. Actual Test Visualization

The wrist is composed of eight carpal bones. These bones are arranged in a proximal row and a distal row, which form joint structures with the radius and ulna. The main joints include the radiocarpal joint and the distal radioulnar joint. These joints allow the wrist to move in multiple directions. The movements include flexion, extension, radial deviation, ulnar deviation, supination, and pronation [26].

The rehabilitation system presented in this article divides the movements into three rehabilitation motions and one standby motion. The system helps patients begin basic rehabilitation exercises as soon as the cast is removed. Early training prevents muscle atrophy and joint stiffness caused by long-term immobilization.

A fully charged system can operate for 14 h. Given that standard daily home rehabilitation routines typically last between 30 and 60 min, a 14 h active runtime provides sufficient battery capacity to support approximately two weeks (14 to 28 days) of continuous daily use without the need for frequent recharging. This level of endurance highly satisfies the practical and convenience requirements for its intended home rehabilitation scenario. The intelligent wearable device detects and judges the specified movements. It provides real-time feedback and sends the recorded data to a database. Medical personnel can review these records and track the patient’s rehabilitation progress.

#### 3.3.1. Radial_Ulnar_Deviation

**Figure 19:**
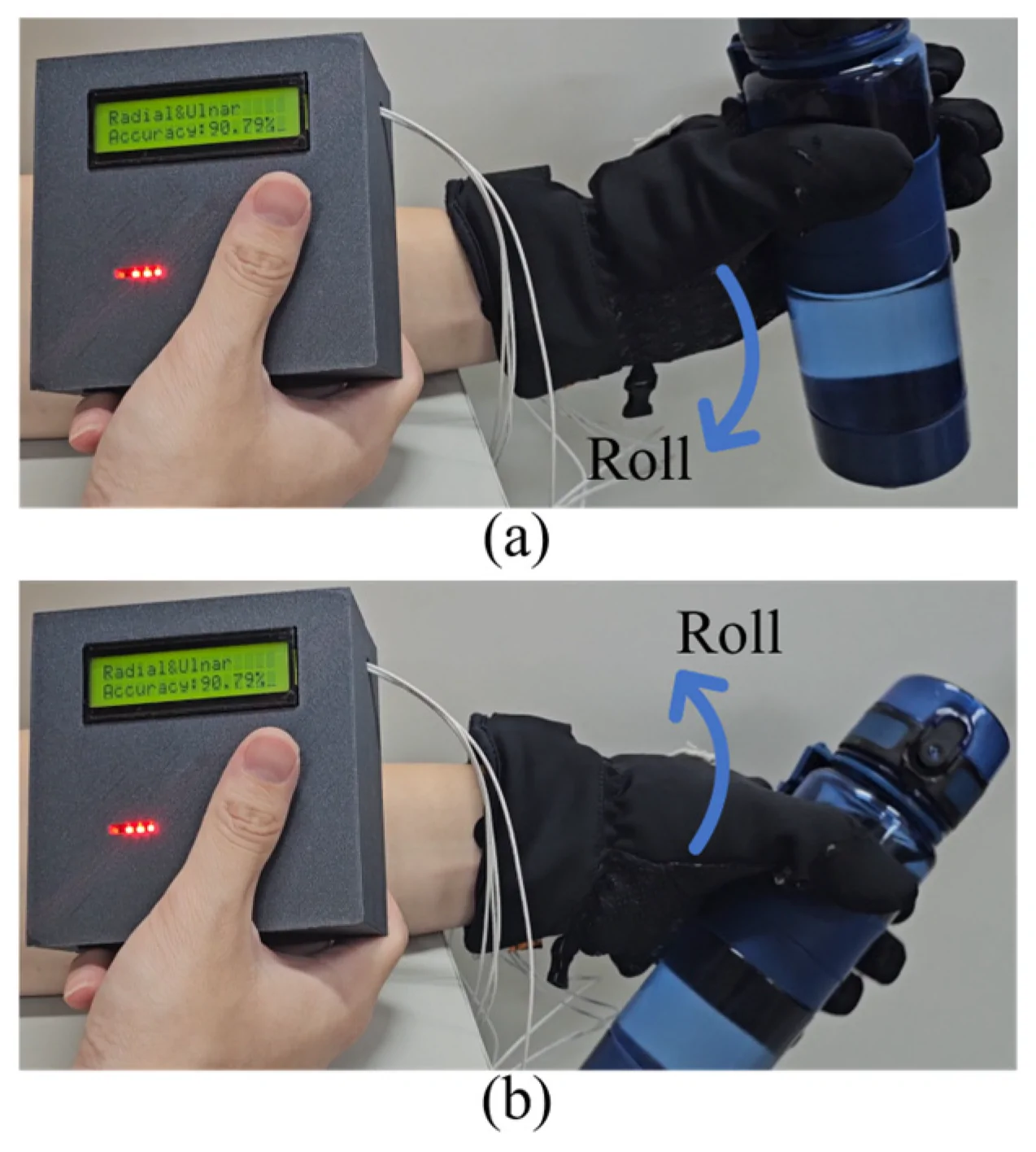
Rehabilitation movement: (**a**) wrist radial deviation (**b**) wrist ulnar deviation.

The main muscle groups involved in this movement are the flexor carpi radialis and flexor carpi ulnaris muscles. Radial and ulnar deviation rehabilitation movements improve wrist flexibility during lateral movement. They also help enhance wrist joint stability in daily activities. Figure 19 illustrates a schematic diagram of radial and ulnar deviation. The system evaluates the accuracy of this movement based on the deviation angles on the radial and ulnar sides.

#### 3.3.2. Flexion_Extension

**Figure 20:**
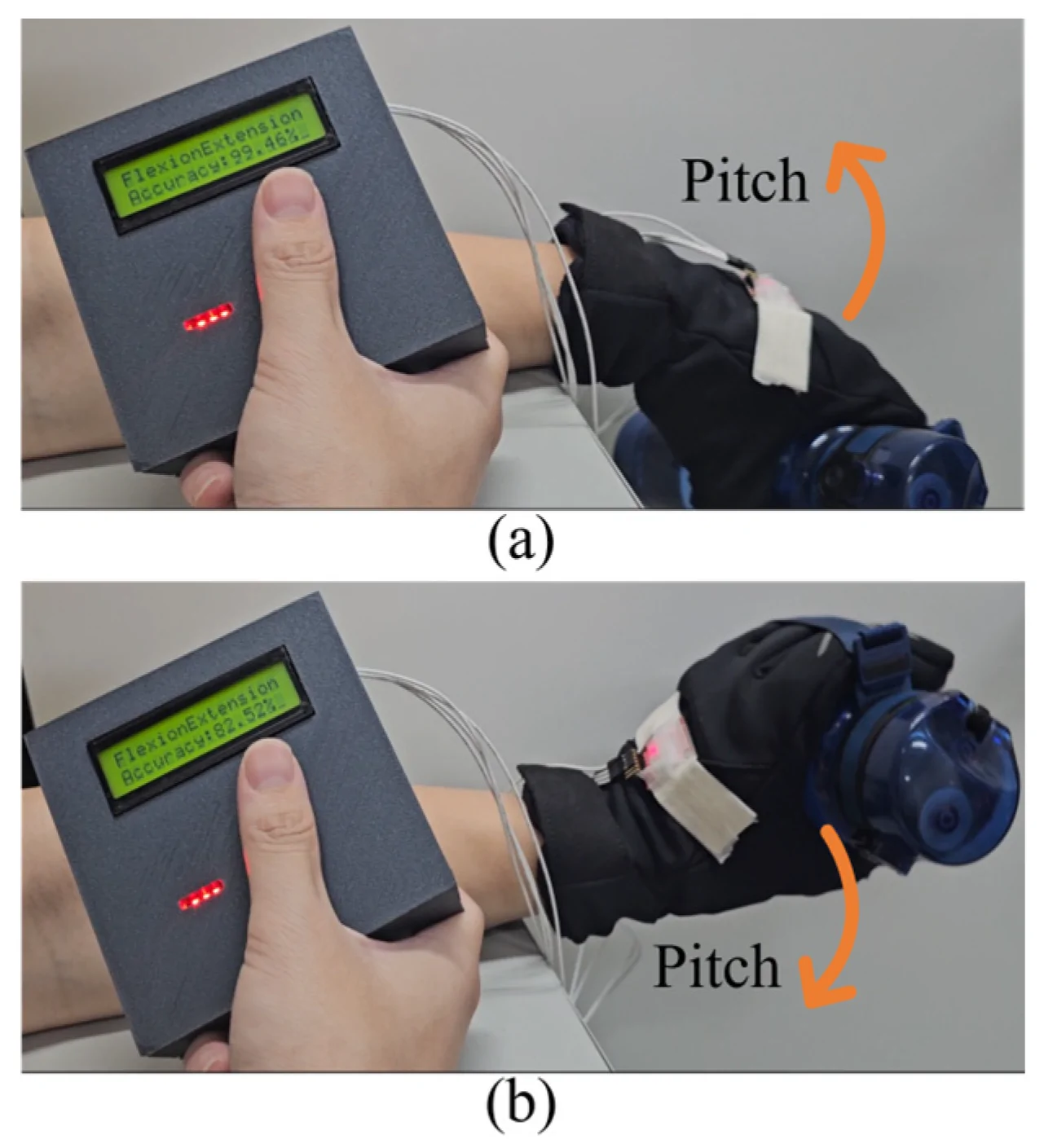
Rehabilitation movement: (**a**) wrist flexion (**b**) wrist extension.

The main muscle groups involved in this movement are the wrist flexors and wrist extensors. Flexion and extension rehabilitation movements help restore the wrist joint’s range of motion. They also help improve wrist function in daily activities such as lifting and gripping. Figure 20 illustrates a schematic diagram of wrist flexion and extension. The system evaluates the accuracy of this movement based on the flexion and extension angles of the wrist.

#### 3.3.3. Pronation_Supination

**Figure 21:**
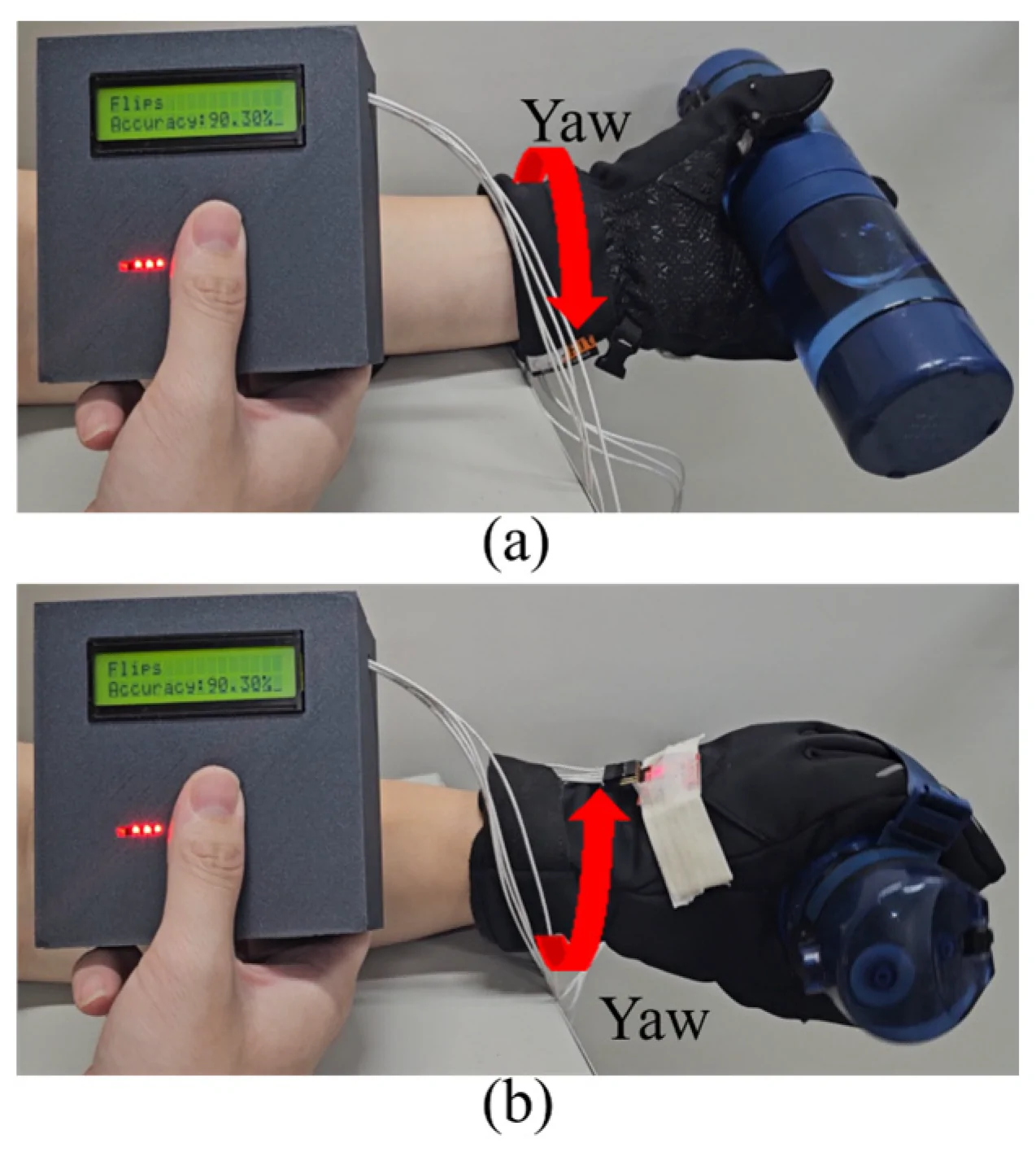
Rehabilitation movement: (**a**) wrist pronation (**b**) wrist supination.

The main muscle groups involved in this movement are the pronator teres and supinator muscles. Pronation and supination rehabilitation movements regain pronation and supination function of the forearms. They also help improve flexibility and coordination when performing daily activities such as opening bottle caps and turning doorknobs. Figure 21 illustrates a schematic diagram of wrist pronation and supination. The system evaluates the accuracy of this movement based on the pronation and supination angles of the forearm.

## 4. Conclusions

This article presents a real-time motion classification and data recording system for autonomous rehabilitation. The system collects motion data using a 9-axis sensor. It converts the data into roll, pitch, and yaw angles as input features for the NanoEdge AI model. This design enables real-time motion classification and recognition. Practical testing shows that a full charge supports up to 14 h of continuous operation. The system features low power consumption and high efficiency. It also includes an LCD display for real-time feedback and a Bluetooth module for database storage. The device can operate independently without a computer connection.

Furthermore, this article provides a web interface and a database for long-term tracking of rehabilitation progress. The system uploads and visualizes historical data. It also offers different access levels for patients and medical personnel. These permissions determine how rehabilitation data are viewed and managed. The overall system architecture includes front-end sensing, real-time inference, Bluetooth communication, and back-end management. The system integrates these functions into a personalized rehabilitation monitoring tool.

## Figures and Tables

**Table 1:** Comparison of wireless communication.

Wireless Communication	Distance	Power Consumption	Scenario
Bluetooth	10–100 m	Low	Wearable device/Short-range transmission
Wi-Fi	50–100 m	High	High-speed transmission/Internet connection
ZigBee	10–100 m	Extremely Low	IoT/Sensor network
LoRa	2–15 km	Low	Long-range low-data transmission

## Data Availability

The original contributions presented in this study are included in the article. Further inquiries can be directed to the corresponding author.

## References

[1] Huang C., Nihey F., Inai T., Kobayashi Y., Fujita K., Yamamoto A., Fujimoto M., Fukushi K., Nakahara K. (2024). Wearable Device and Algorithm for Fall Prevention: Monitoring Fall Risk Using Foot Motion Measured by an In-Shoe Motion Sensor. IEEE Trans. Instrum. Meas..

[2] Tran T.N.H.T., Le L.H., Ta D. (2022). Ultrasonic Guided Waves in Bone: A Decade of Advancement in Review. IEEE Trans. Ultrason. Ferroelectr. Freq. Control.

[3] Colles Fracture. https://my.clevelandclinic.org/health/diseases/21860-colles-fracture.

[4] Caiti G., Dobbe J.G.G., Strackee S.D., Strijkers G.J., Streekstra G.J. (2020). Computer-Assisted Techniques in Corrective Distal Radius Osteotomy Procedures. IEEE Rev. Biomed. Eng..

[5] Marrugo-Tobon D., Romero-Mercado C., Mendoza-Vanegas K., Magre-Colorado L., Rios Y.Y. (2020). Ergonomic Portable Measuring System Designed to Assist Patients with Metacarpal Injuries. Proceedings of the 9th 2020 IX International Congress of Mechatronics Engineering and Automation.

[6] Zhang C., Huang M.Z., Kehs G.J., Braun R.G., Cole J.W., Zhang L.-Q. (2021). Intensive In-Bed Sensorimotor Rehabilitation of Early Subacute Stroke Survivors With Severe Hemiplegia Using a Wearable Robot. IEEE Trans. Neural Syst. Rehabil. Eng..

[7] Tamantini C., Cordella F., Lauretti C., di Luzio F.S., Campagnola B., Cricenti L., Bravi M., Bressi F., Draicchio F., Sterzi S. (2023). Tailoring Upper-Limb Robot-Aided Orthopedic Rehabilitation on Patients’ Psychophysiological State. IEEE Trans. Neural Syst. Rehabil. Eng..

[8] Liao Y., Vakanski A., Xian M. (2020). A Deep Learning Framework for Assessing Physical Rehabilitation Exercises. IEEE Trans. Neural Syst. Rehabil. Eng..

[9] Xu W., Xiang D., Wang G., Liao R., Shao M., Li K. (2022). Multiview Video-Based 3-D Pose Estimation of Patients in Computer-Assisted Rehabilitation Environment (CAREN). IEEE Trans. Hum.-Mach. Syst..

[10] Lv Z., Singh A.K. (2024). Edge-Cloud-Based Wearable Computing for Automation Empowered Virtual Rehabilitation. IEEE Trans. Autom. Sci. Eng..

[11] He Q., Xi Z., Feng Z., Teng Y., Ma L., Cai Y., Yu K. (2025). Telemedicine Monitoring System Based on Fog/Edge Computing: A Survey. IEEE Trans. Serv. Comput..

[12] He C., Xiong C.-H., Chen Z.-J., Fan W., Huang X.-L., Fu C. (2021). Preliminary Assessment of a Postural Synergy-Based Exoskeleton for Post-Stroke Upper Limb Rehabilitation. IEEE Trans. Neural Syst. Rehabil. Eng..

[13] Rho E., Lee H., Lee Y., Lee K.-D., Mun J., Kim M., Kim D., Park H.-S., Jo S. (2024). Multiple Hand Posture Rehabilitation System Using Vision-Based Intention Detection and Soft-Robotic Glove. IEEE Trans. Ind. Inform..

[14] Berta R., Kobeissi A., Bellotti F., De Gloria A. (2021). Atmosphere, an Open Source Measurement-Oriented Data Framework for IoT. IEEE Trans. Ind. Inform..

[15] Xu B., Kong L., Wen G., Pecht M.G. (2021). Protection Devices in Commercial 18650 Lithium-Ion Batteries. IEEE Access.

[16] Yao X.-Y., Kong L., Pecht M.G. (2020). Reliability of Cylindrical Li-ion Battery Safety Vents. IEEE Access.

[17] Majumder S., Deen M.J. (2020). A Robust Orientation Filter for Wearable Sensing Applications. IEEE Sens. J..

[18] Gong C., Zhao F., Liao Z., Tao T., Wang X., Mei X. (2024). Simple-Rotation Angle/Axis Representations Based Second-Order Impedance Control. IEEE Robot. Autom. Lett..

[19] Kim J.-H., Park S.-S., Park K.-K., Ryoo C.-K. (2021). Quaternion Based Three-Dimensional Impact Angle Control Guidance Law. IEEE Trans. Aerosp. Electron. Syst..

[20] Chen X., Xie Z., Eun Y., Bettens A., Wu X. (2023). An Observation Model From Linear Interpolation for Quaternion-Based Attitude Estimation. IEEE Trans. Instrum. Meas..

[21] Rong H., Peng C., Chen Y., Lv J., Zhu Y., Zou L. (2025). A Fast Complementary Filter for Avoiding the Magnetometer Measurements From Influencing the Calculation of Pitch and Roll. IEEE Sens. J..

[22] Kim S., Kim M. (2023). Rotation Representations and Their Conversions. IEEE Access.

[23] Tayeb F., Chihaoui H., Filali F. (2023). Bluetooth-Based Vehicle Counting: Bridging the Gap to Ground-Truth With Machine Learning. IEEE Access.

[24] Skanda C., Srivatsa B., Premananda B. (2022). Secure Hashing using BCrypt for Cryptographic Applications. Proceedings of the 1st 2022 IEEE North Karnataka Subsection Flagship International Conference (NKCon).

[25] Liu J., Wan G., Wang S., Deng P., Su Z., Li C., Mu Y., Yin Y. (2023). Visually Enhanced Parallel Coordinates Plot With Two-Dimensional Kernel Density Scatter Plots. IEEE Access.

[26] Functional Anatomy of the Wrist. https://www.physio-pedia.com/Functional_Anatomy_of_the_Wrist?utm_source=physiopedia&utm_medium=search&utm_campaign=ongoing_internal#editor.

